# Uncovering the mode of action of engineered T cells in patient cancer organoids

**DOI:** 10.1038/s41587-022-01397-w

**Published:** 2022-07-25

**Authors:** Johanna F. Dekkers, Maria Alieva, Astrid Cleven, Farid Keramati, Amber K. L. Wezenaar, Esmée J. van Vliet, Jens Puschhof, Peter Brazda, Inez Johanna, Angelo D. Meringa, Heggert G. Rebel, Maj-Britt Buchholz, Mario Barrera Román, Amber L. Zeeman, Sam de Blank, Domenico Fasci, Maarten H. Geurts, Annelisa M. Cornel, Else Driehuis, Rosemary Millen, Trudy Straetemans, Mara J. T. Nicolasen, Tineke Aarts-Riemens, Hendrikus C. R. Ariese, Hannah R. Johnson, Ravian L. van Ineveld, Froso Karaiskaki, Oded Kopper, Yotam E. Bar-Ephraim, Kai Kretzschmar, Alexander M. M. Eggermont, Stefan Nierkens, Ellen J. Wehrens, Henk G. Stunnenberg, Hans Clevers, Jürgen Kuball, Zsolt Sebestyen, Anne C. Rios

**Affiliations:** 1 Hubrecht Institute, Royal Netherlands Academy of Arts and Sciences and University Medical Center Utrecht, Utrecht, the Netherlands; 2 Princess Máxima Center for Pediatric Oncology, Utrecht, the Netherlands; 3 Oncode Institute, Utrecht, the Netherlands; 4 Center for Translational Immunology, University Medical Center Utrecht, Utrecht University, Utrecht, the Netherlands; 5 Microbiome and Cancer Division, German Cancer Research Center, Heidelberg, Germany; 6 Department of Hematology, University Medical Center Utrecht, Utrecht University, Utrecht, the Netherlands; 7 Mildred Scheel Early Career Center for Cancer Research Würzburg, University Hospital Würzburg, MSNZ/IZKF, Wurzburg, Germany; 8 University Medical Center Utrecht, Utrecht, the Netherlands; 9 Comprehensive Cancer Center München, Munich, Germany; 10 Pharma, Research and Early Development, F. Hoffmann-La Roche Ltd, Basel, Switzerland

**Keywords:** Biological techniques, Immunology, Cancer imaging, Immunotherapy

## Abstract

Extending the success of cellular immunotherapies against blood cancers to the realm of solid tumors will require improved in vitro models that reveal therapeutic modes of action at the molecular level. Here we describe a system, called BEHAV3D, developed to study the dynamic interactions of immune cells and patient cancer organoids by means of imaging and transcriptomics. We apply BEHAV3D to live-track >150,000 engineered T cells cultured with patient-derived, solid-tumor organoids, identifying a ‘super engager’ behavioral cluster comprising T cells with potent serial killing capacity. Among other T cell concepts we also study cancer metabolome-sensing engineered T cells (TEGs) and detect behavior-specific gene signatures that include a group of 27 genes with no previously described T cell function that are expressed by super engager killer TEGs. We further show that type I interferon can prime resistant organoids for TEG-mediated killing. BEHAV3D is a promising tool for the characterization of behavioral-phenotypic heterogeneity of cellular immunotherapies and may support the optimization of personalized solid-tumor-targeting cell therapies.

## Abstract (web summary)

The dynamics and molecular mechanisms of T cell therapies are probed in cancer organoids.

## Main

Single-cell analyses are providing unprecedented opportunities to analyze the complexity of biological systems^1^. However, they are restricted to providing a snapshot of cellular processes, lacking analysis of dynamic behavior inherent to cell function. Therefore, the development of technologies that address individual cell dynamics will be essential for understanding cellular behavior and how it relates to function. Immune cells engineered to kill tumor cells represent such dynamic cell populations with increasing clinical importance^2^. Successes of T cell therapies for hematological malignancies have sparked efforts for translation to solid tumors, but efficacy has so far been limited^3^. This poses a clear need for better understanding of the mechanism of action of cellular therapies to optimize treatment design.

Various T cell therapy concepts are being developed to target cancer, including chimeric antigen receptor (CAR)^4^ and conventional T cell receptor (TCR)^5^ T cell therapies, as well as αβ T cells engineered to express a γδ TCR (TEGs)^6–10^, endowing cancer-recognizing properties through metabolic sensing^10–12^. Because of their ability to recapitulate important characteristics of the original tumor specimen^13^, including patient-specifc responses to treatment^14–18^, there is a growing interest in the use of patient-derived organoids (PDOs) to model immunotherapy function^19–23^. At the same time, imaging has proved a powerful approach to characterizayion of the spatial cellular organization and tissue dynamics in these three-dimensional (3D) structures^24–28^, including CAR T cell treatment efficacy in immuno-organoid cocultures^23^. However, imaging has not yet been used to probe in depth the solid-tumor-targeting dynamics of cellular immunotherapy with PDOs, which could generate critical insight into their mode of action in a patient-specific manner that could be exploited towards improved therapy design. Therefore, here we combined organoid and 3D imaging technology for the analysis of functional single-cell behavior integrated with transcriptomic profiling, to decipher and manipulate the solid-tumor-targeting strategy of engineered immune cells (Supplementary Video 1).

## Results

### 3D live-tracked TEG targeting efficacy

**Fig. 1:**
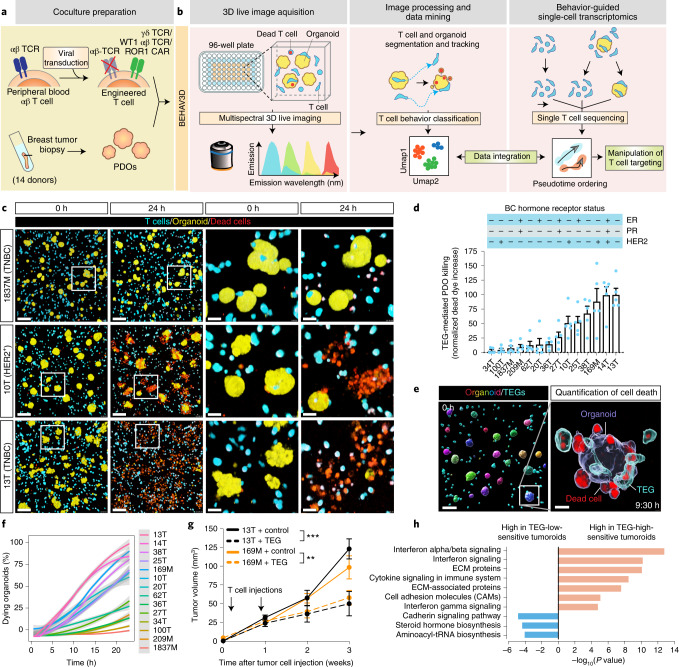
TEG efficacy across organoids of multiple BC subtypes detected by multispectral 3D live imaging and in vivo TEG targeting. **a**,**b**, Schematic representation of TEG generation and coculture with PDOs (**a**) and of the BEHAV3D platform (**b**). Cocultures of organoids and TEGs were imaged using 3D microscopy, followed by segmentation and tracking of organoids and T cells and subsequent behavior classification. Pseudotime ordering was used to integrate behavioral data. **c**, 3D multispectral images of breast PDO cultures (yellow) showing low (1837M), intermediate (10T) and high (13T) killing by TEGs (blue) at the indicated time points of imaging. Scale bars, 100 µm (two left-hand columns) and 30 µm (two right-hand columns). **d**, Quantification of killing of organoids derived from 14 patients with BC following 24-h coculture with TEGs, by 3D live-cell imaging. Data were corrected for control LM1 T cell responses (*n* = 4 independent experiments, mean ± s.e.m. TNBC, triple-negative breast cancer; ER, estrogen receptor; PR, progesterone receptor. **e**, 3D image of organoids and T cells; enlarged section showing the presence of dead cell dye (red) in a single organoid (transparent purple rendering) and TEGs (transparent blue rendering) at the indicated time of coculture. Scale bars, 100 µm (left) and 30 µm (right). **f**, Quantification of the percentage of dying single organoids (of total) over time for each PDO cocultured with TEGs (*n* = 4 independent experiments, mean ± s.e.m.). **g**, Quantification of tumor volume over time generated by subcutaneous transplantation of 13T (black) or 169M organoids (orange). Animals received two injections of either TEGs (dashed line) or control TEG011 cells (control, solid line) at the indicated time points (*n* = 10 mice for 13T and *n* = 15 for 169M, mean ± s.e.m.). Two-way ANOVA with repeated measures: 13T/TEG versus 13T/control, *P* < 0.0001 (***); 169M/TEG versus 169M/control, *P* = 0.0016 (**). **h**, Gene Ontology (GO) enrichment analysis of DEGs between the six highest versus six lowest TEG-sensitive organoid cultures from **d**. **c**,**e**, Images representative of *n* = 4 independent experiments.

**Extended Data Fig. 1:**
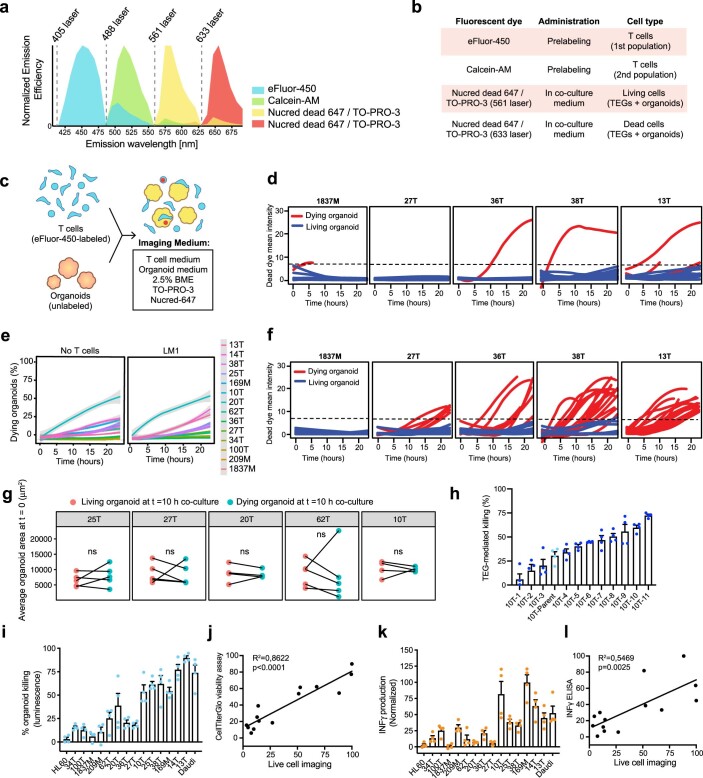
Multi-spectral 3D imaging quantification of organoid killing. (**a**) Emission spectra of the indicated fluorescent real-time cell dyes separately imaged by multispectral imaging with the lambda mode using the indicated lasers. (**b**) Overview of fluorescent real-time cell dyes for labelling the indicated cell types. (**c**) Schematic representation of the co-culture setup. (**d**) Quantification of death of individual PDOs in the presence of control TEGs expressing a mutated Vψ9/V82 TCR (LM1s). (**e**) Quantification of the percentage of dying single organoids (% of total) over time for each PDO co-cultured with LM1 control TEGs (right panel) or in the absence of T cells (left panel) (n = 4 independent experiments; mean). (**f**) Quantification of death of individual PDOs in the presence of TEGs. (**g**) Comparison of average size of PDOs (t = 0 of TEG co- culture) that were either dying or alive at 10 h of co- culture with TEGs for the indicted PDO lines. Data corrected for control LM1 T cell responses. n = 4 (20 T and 10 T) or 5 (25 T, 27 T, 62 T) independent experiments. Two-tailed unpaired t test: NS = p > 0.05. (**h**) Quantification of killing of 11 10 T PDO clonal lines as well as the parental culture using a CellTiter-Glo® viability assay, upon overnight co-culture of organoids with TEGs in the presence of pamidronate, (n = 3 (10T-1 and 10T-2) or 4 (all other clones) independent experiments; mean ± s.e.m.). (**i-l**) Quantification of PDO targeting using a CellTiter-Glo® viability assay (i) or INFψ ELISA assay (k), upon 24 h co-culture of organoids with TEGs in the presence of pamidronate, and Pearson correlation plots between the outcomes of live cell imaging compared to CellTiter-Glo® measured viability (j). (F = 75.05, DFn=1, DFd=12, 95% CI [0.5184, 0.8668], p < 0.0001) and INFψ ELISA (l) (F = 14.49, DFn=1, DFd=12, 95% CI [0.257,0.9452], p = 0.0025). Data corrected for control LM1 T cell responses. (n = 4 independent experiments; mean ± s.e.m.). (d,f: Single organoids that crossed the mean dead cell dye intensity threshold of 7 (dashed lines) are considered dying (red lines)).

We devised BEHAV3D, a multispectral, 3D image-based platform, to live-track the efficacy and mode of action of cellular immunotherapy for ~60 human cancer organoid cultures simultaneously (Fig. 1a–c, Supplementary Video 1, Extended Data Fig. 1a–c and Methods). Applied to an extensive and well-characterized breast cancer (BC) PDO biobank^29^ and cancer metabolome-sensing TEGs, we detected a high variation of TEG-mediated killing efficacy in cultures derived from 14 patients with BC (Fig. 1d and Supplementary Table 1) and different targeting kinetics over time (Fig. 1e,f and Extended Data Fig. 1d–f), with percentages of dying PDOs ranging from near 0 (for example, 34T) to 100 (for example, 13T) (Fig. 1f). This variation in PDO killing kinetics was also observed between single organoids in the same PDO culture (Extended Data Fig. 1f), and we show that this is not related to differences in organoid size at the start of coculture (Extended Data Fig. 1g). Instead, by subcloning of 10T PDOs, we demonstrate that each clone displayed an individual level of targeting that was stably maintained over multiple passages (Extended Data Fig. 1h), suggesting an intrinsic biological diversity in sensitivity. This, furthermore, demonstrates that BEHAV3D can adequately capture such functional heterogeneity within PDO cultures, as well as between patients. Pearson correlation analysis between imaging data and a commonly used cell viability assay (Extended Data Fig. 1i,j) or interferon gamma (IFN-γ) secretion (Extended Data Fig. 1k,l), confirmed the robustness of our imaging quantification method. Among the six highest TEG-sensitive BC PDO cultures (>50% dying organoids; Fig. 1f), we noted cultures derived from primary BC of distinct subtypes, as well as a metastasis-derived sample (Fig. 1d,f). In addition, TEGs controlled the growth of PDO-derived breast tumor in vivo in mouse xenograft models (Fig. 1g). Together, this provides evidence in favor of the clinical potential of TEG against solid tumors and, specifically, pan-targeting of BC, albeit with variation in responsiveness among individual donors.

### PDO inflammatory features are associated with TEG sensitivity

**Extended Data Fig. 2:**
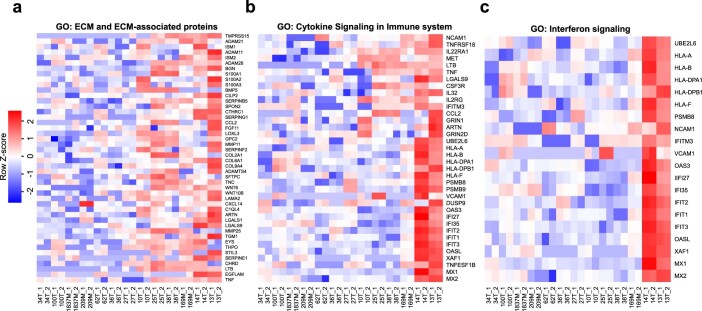
GO terms associating with PDO sensitivity to TEGs. (**a-c**) Heatmap showing normalized gene expression (Row Z-score) for the indicated PDOs harvested at two different time points in culture (experimental replicates ‘_1’ and ‘_2’). GO terms ‘extracellular matrix (ECM) and ECM- associated proteins’ (a), ‘cytokines signaling in immune system’ (b) and ‘interferon signaling’ (c) are presented, which were identified in the gene ontology enrichment analysis of differentially expressed genes between the six highest versus six lowest TEG-sensitive organoid cultures from Fig. 1h.

Bulk RNA sequencing (RNA-seq) of BC PDOs revealed differentially expressed genes (DEGs) between the six lowest versus the six highest TEG-sensitive PDO cultures (Supplementary Table 2), related to upregulated cadherin signaling and steroid biosynthesis pathways in TEG-insensitive cultures, whereas both cytokine signaling and extracellular matrix (ECM) organization, correlated with high sensitivity to TEG therapy (Fig. 1h and Extended Data Fig. 2a–c). The highest association was found between TEG killing and type 1 interferon (IFN-I) signaling genes, including *MX1, IFIT1, OASL* and *XAF1*, which were highly expressed, especially in the two highest TEG-sensitive PDO cultures, 14T and 13T (Fig. 1h and Extended Data Fig. 2c). Thus, PDOs maintain tumor-specific inflammatory features in culture, highlighting their utility for modeling cellular immunotherapy responses in a patient-specific manner.

### TEGs display high diversity in behavior and killing potential

**Fig. 2:**
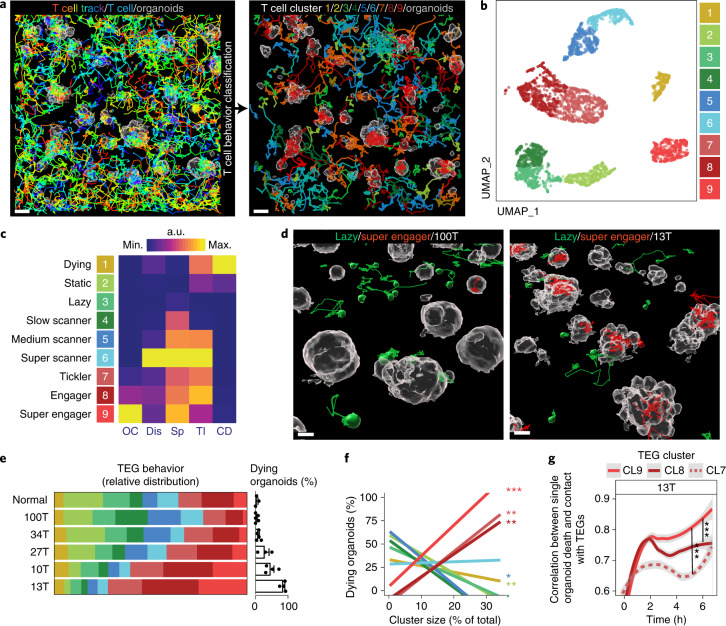
TEGs exposed to PDOs display high diversity in their behavior with distinct killing potential. **a**, Image of automated tracking of each TEG (left, 10-h tracks are rainbow colored for time). Tracks were classified according to TEG behavior and back-projected in the image (right, color coded by cluster). Scale bars, 50 µm. Representative of *n* = 11 independent experiments. **b**, UMAP plot showing nine color-coded clusters identified by unbiased multivariate time series dynamic time-warping analysis. Each data point represents one T cell track of 3.3 h. See Supplementary Table 8 for conditions and replicates included. **c**, Heatmap depicting relative values of T cell features indicated for each cluster, named according to their most distinct characteristics. a.u., Arbitrary units in respect to maximal and minimal values for each feature. OC, organoid contact; Dis, square displacement; Sp, speed; TI, T cell interactions; CD, cell death. **d**, 3D-rendered images of 100T (low-targeting, left) and 13T (high-targeting, right) organoids (gray) and TEGs, with 3.3-h tracks belonging to lazy (green) and super engager (red) clusters. Scale bars, 20 µm. Representative of *n* = 5 independent experiments. **e**, Behavioral cluster distribution of TEGs cocultured with the indicated PDOs and a normal organoid culture (left), in relation to their killing capacity (right, bar graph), represented as the percentage of dying organoids (percentage of total); *n* ≥ 3 independent experiments, mean ± s.e.m. *X*^2^-test, *P* = 1.132 × 10^–8^. **f**, Pearson correlation between behavior cluster (CL) size and percentage of dying organoids represented in **d**. CL9, *P* = 0.00006 (***); CL8, *P* = 0.009 (**); CL7, *P* = 0.006 (**); CL5, *P* = 0.014 (*); CL4, *P* = 0.022 (*); CL2, *P* = 0.0019 (**) (mean). See Supplementary Table 8 for test statistics and replicates included. **g**, Change in correlation between 13T organoid death dynamics (measured as increase in dead cell dye) and cumulative contact with TEGs (from CL7–9). Data presented as mean correlation per time point of all single organoids (*n* = 4 independent experiments). Linear mixed model fitting with each experimental replicate as a random effect: C9 versus C8, *P* = 5.19 × 10^–6^ (***); C9 versus C7, *P* < 2 × 10^–16^ (***).

**Extended Data Fig. 3:**
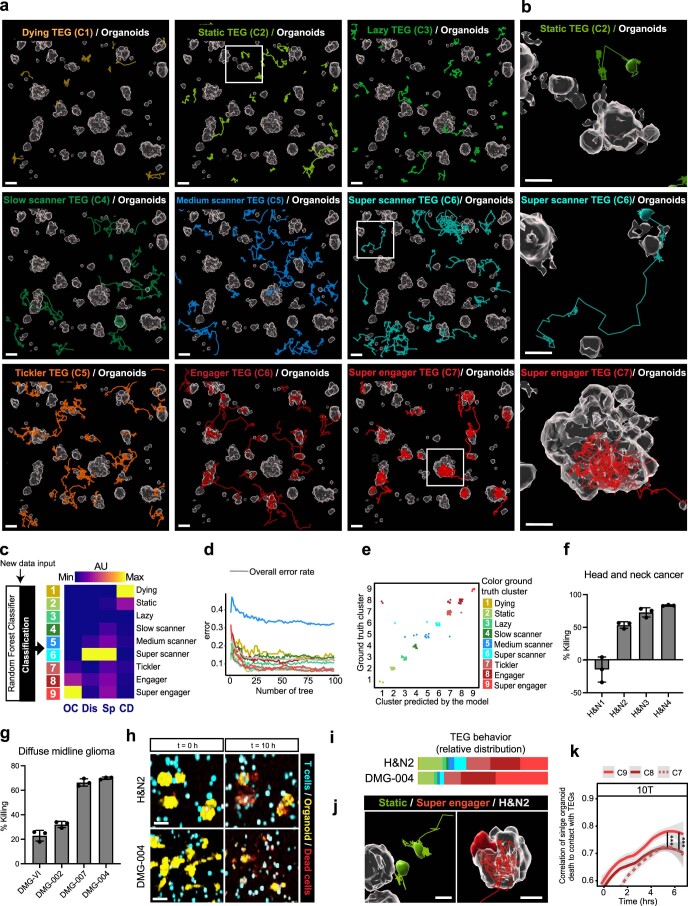
Properties of the 9 TEG behavioral clusters, random forest classification and head and neck cancer and diffuse midline glioma PDO targeting. (**a,b**) Representative multispectral overview images (a; scale bars, 50 µm) and enlarged sections for clusters of interest (b; scale bars, 20 µm) of 13 T organoids co- cultured with TEGs classified into 9 different behavioral clusters. n = 11 independent experiments. (**c**) Schematic representation of the Random Forest classification pipeline and the resulting heatmap showing relative intensity values of T cell features indicated for each cluster resulting from the classification of the experiment in Fig. 2c. (OC, organoid contact; Dis, square displacement; Sp, speed; TI, T-cell interaction; CD, cell death) (**d,e**) Error rate of the training data per cluster and overall for all trees (d) and correlation plot between ground truth cluster classification and predicted cluster classification (e). Color represents ground truth cluster. (**f,g**) Quantification of head and neck cancer (H&N) PDO (f) or diffuse midline glioma (DMG) PDO (g) targeting using a CellTiter-Glo® viability assay upon overnight co- culture with TEGs in the presence of pamidronate. Data corrected for control LM1 T cell responses. (n = 3 independent wells, representative graph of n = 3 independent experiments; mean ± s.d.). (**h**) Images of H&N cancer & DMG PDO cultures (yellow) showing killing by TEGs (blue) at the indicated time points of imaging. Dead cells in red. Scale bars, 50 µm. (**i**) Behavioral cluster distribution of TEGs co-cultured with the indicated PDOs. χ^2^ test; p = 1.132e-08. (**j**) Representative multispectral images of H&N2 PDOs (rendered in grey) co-cultured with TEGs classified as *static* (C2; green) or *super engager* (C9; red), Scale bars, 15 µm. (**k**) Change in correlation between 10 T organoid death dynamics (measured as increase in dead cell dye) and cumulative contact with TEGs (from behavior clusters 7-9). Data is represented as mean correlation per timepoint of all single organoids (n = 4 independent experiments). Linear mixed model fitting with each experimental replicate as a random effect: C9 vs C8, p < 2e-16; C9 vs C7, p < 2e-16. (h-j: representative data of n = 3 independent experiments).

BEHAV3D implements single-immune cell tracking in a 3D space over time and behavioral classification (Figs 1b and 2a and Supplementary Video 1), revealing that, when exposed to BC PDOs, TEGs could be separated into nine subpopulations with unique behavioral patterns (Fig. 2b–d and Extended Data Fig. 3a,b). Patterns varied from inactive behaviors (dying, static and lazy) to active motility (slow scanner, medium scanner and super scanner) and organoid engagement (tickler, engager and super engager), thus demonstrating a high level of behavioral heterogeneity. We captured this behavioral single-cell landscape in a classifier (Extended Data Fig. 3c–e), allowing us to interrogate and predict engineered T cell behavior under other coculture conditions by tracking >150,000 T cells in total. First, we investigated targeting of different solid-tumor subtypes beyond BC and detected TEG targeting of PDOs from head and neck cancer (3/4 PDOs killed with 50–90% killing efficacy), as well as in patients with diffuse midline glioma (DMG) (4/4 PDOs killed with 20–90% killing efficacy; Extended Data Fig. 3f–h). We observed comparable behavioral diversity of TEGs, as seen for BC targeting, including static and super engager behavior (Extended Data Fig. 3i,j). This not only further supports the broad solid-tumor-targeting efficacy of TEGs, but also shows that extensive behavioral heterogeneity of TEGs is universally present among different solid-tumor PDO cocultures.

Next we used our behavioral classifier (Extended Data Fig. 3c–e) to predict TEG behavior when cocultured with BC PDOs showing varying TEG sensitivity (34T, 100T, 27T, 10T or 13T; Fig. 1f), as well as an organoid culture derived from normal breast tissue showing only minimal death when cultured with TEGs (Fig. 2e). For each PDO culture, TEGs displayed unique distributions of behavioral signatures (Fig. 2e) and higher organoid killing associated with an increase in tumor engagement (tickler, engager and super engager), while static, lazy and medium scanner behavior decreased (Fig. 2f). Correlation between single-organoid dying dynamics and TEG engagement over time revealed that organoids contacted by super engagers, as compared with other organoid-engaging clusters, had the highest chance of being killed (Fig. 2g and Extended Data Fig. 3k). This indicates that effective killing by TEGs relies on prolonged organoid contact, a key feature of super engagers (48 ± 8 min h^–1^; mean ± s.d.).

### Behavioral differences detected between engineered T cell therapies

**Fig. 3:**
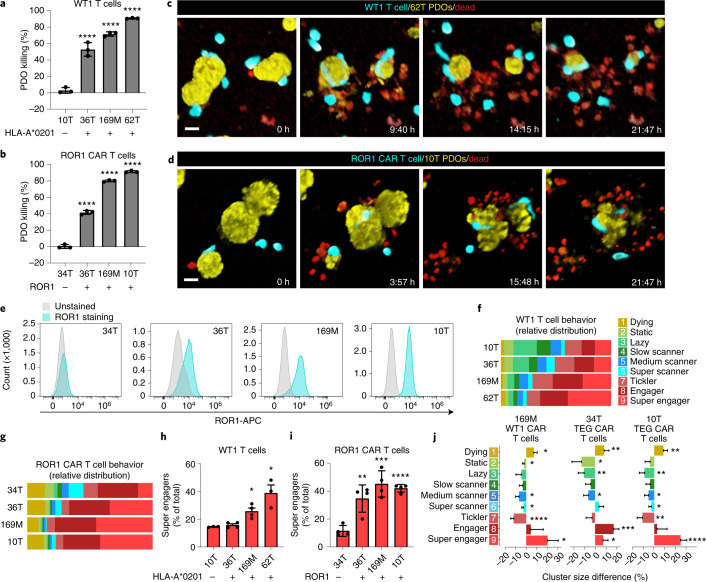
Behavioral heterogeneity of TCR and CAR T cell therapies targeting BC PDOs. **a**,**b**, Quantification of BC PDO viability using CellTiter-Glo following overnight coculture of PDOs with WT1 T cells (**a**) or ROR1 CAR T cells (**b**). **a**,**b**, One-way ANOVA followed by Dunnett’s correction: 10T versus 36T, *P* < 0,0001 (****); 10T versus 169M, *P* < 0,0001 (****); 10T versus 62T, *P* < 0,0001 (****) (**a**); 34T versus 36T, *P* < 0,0001 (****); 34T versus 169M, *P* < 0,0001 (****); 34T versus 10T, *P* < 0.0001 (****) (**b**). Data corrected for untransduced T cell responses (mean ± s.d.). **c**,**d**, 3D multispectral images of BC PDO cultures (yellow) showing killing by WT1 T cells (blue, **c**) or ROR1 CAR T cells (blue, **d**) at the indicated time points of imaging. Dead cells depicted in red. Scale bars, 30 µm. **e**, FACS histogram plots showing ROR1 expression in the indicated breast cancer PDO cultures (blue) compared with unstained control (gray). **f**,**g**, Behavioral cluster distribution of WT1 T cells (**f**) and ROR1 CAR T cells (**g**) cocultured with the indicated BC PDOs (mean ± s.e.m.). *X*^2^-test, *P* < 0,0001. **h**,**i**, Super engager (CL9) cluster size (%) of total for WT1 T cells (**h**) and ROR1 CAR T cells (**i**). **h**, One-way ANOVA with Dunnett’s correction: 10T versus 169M, *P* = 0,0501; 10T versus 62T, *P* = 0.0006; 34T versus 36T, *P* = 0.0018; 34T versus 169M, *P* < 0.0001; 34T versus 10T, *P* = 0.0002 (mean ± s.d.). **j**, Behavioral cluster size difference (%) between TEGs and CAR T cells cocultured with 34T (middle) or 10T (right), or between WT1 T cells and CAR T cells cocultured with 169M PDOs (left) (mean ± s.d.). Welch’s two-sided *t*-test: 169M: CL1, *P* = 0.015; CL2, *P* = 0.041; CL5, *P* = 0.023; CL6, *P* = 0.047; CL7, *P* = 9.94 × 10^–4^; CL9, *P* = 0.012. 34 T: CL1, *P* = 0.004; CL2, *P* = 0.016; CL3, *P* = 0.003; CL5, *P* = 0.012; CL8, *P* = 0.0004; CL9, *P* = 0.037. 10T: CL1, *P* = 0.0014; CL3, *P* = 0.0045; CL5, *P* = 0.014; CL6, *P* = 0.025; CL7, *P* = 0.001; CL9, *P* = 1.16 × 10^–5^. **a**–**e**, Representative of *n* = 3 independent experiments; **f**–**j**, *n* = 3–6 independent experiments; see Supplementary Table 8 for value of *n* per condition.

We next applied BEHAV3D to evaluate two alternative T cell immunotherapy concepts: (1) T cells engineered with a conventional αβ TCR that targets tumor cells through recognition of the cancer-specific Wilms tumor antigen-1 (WT1) peptide presented on HLA-A*0201 (ref. ^30^), and (2) T cells engineered to express a CAR targeting the tumor-expressed receptor tyrosine kinase-like orphan receptor 1 (ROR1) antigen^4^. We found that WT1 and ROR1 CAR T cells effectively killed various BC PDOs (Fig. 3a–d) while, as expected, a HLA-A*0201^–^ (10T) and ROR1^–^ line (34T) (Fig. 3e) were not killed. T cell behavioral analysis showed similar detection of nine behavioral clusters, as identified for TEGs (Fig. 3f,g), and a substantial larger proportion of the super engager cluster in (highly) targeted PDOs compared with nontargeted PDOs (Fig. 3h,I and Supplementary Video 1). Finally, by comparing therapies that killed certain PDOs at similar efficacy, BEHAV3D uncovered behavioral differences between the different engineered T cells (Figs. 1d and 3a,b). CAR T cells were enriched in active behaviors, including super engager behavior, while showing an increased death rate compared with both WT1 T cells and TEGs (Fig. 3j). Together, these data demonstrate the broad applicability of the BEHAV3D pipeline to various cellular immunotherapies, with the important opportunity to compare and correlate T cell behavior to tumor targeting for identification of the most potent engineered T cells.

### Serial killing capability of super engager CD8^+^ TEGs

**Fig. 4:**
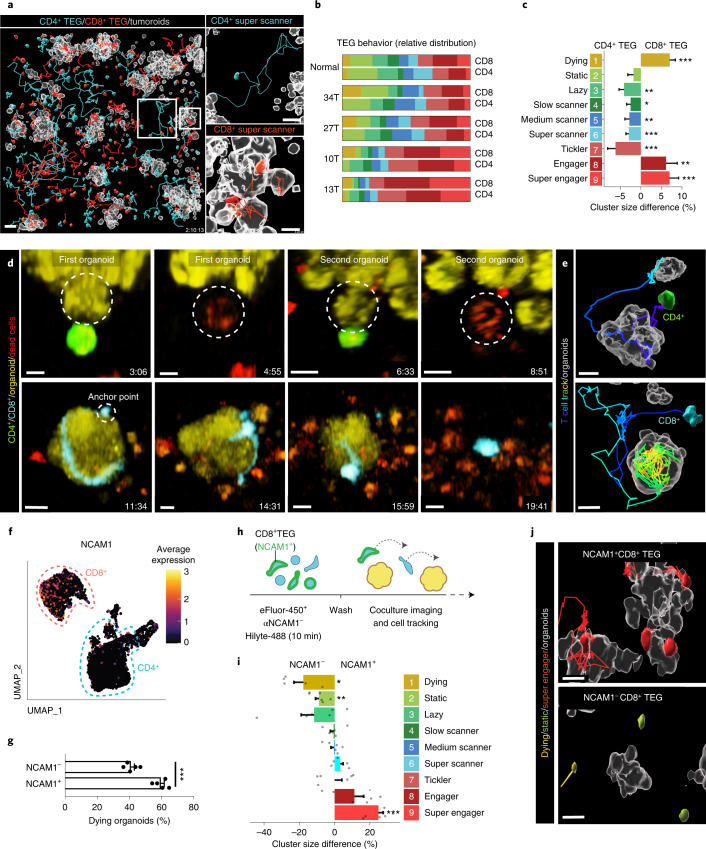
Unique targeting features of TEG subpopulations and serial killer potential. **a**, Images of CD4^+^ (blue) and CD8^+^ (red) TEGs and their full tracks (up to 10 h) cocultured with 13T organoids (gray surface rendering at *t* = 0). Scale bars, 50 µm (main image), 30 µm (zoomed-in images). **b**, Relative behavioral cluster distribution of TEGs cocultured with various organoids. **c**, Behavioral cluster size difference (%) between CD4^+^ and CD8^+^ TEGs cocultured with the indicated organoid cultures from **b** (*n* = 33 wells pooled from the five organoid cultures shown in **b**; see Supplementary Table 8 for replicate specifics; mean ± s.e.m.). Linear regression model fitting with each well as a random effect: CL9, *P* = 7.52 × 10^–6^ (***); CL8, *P* = 0.0034 (**); CL7, *P* = 0.00018 (***); CL6, *P* = 0.000023 (***); CL5, *P* = 0.0062 (**); CL4, *P* = 0.01 (*); CL3, *P* = 0.001 (**); CL1, *P* = 3.01 × 10^–6^ (***). **d**, A CD4^+^ TEG killing a 13T tumor cell in a first organoid and a second tumor cell in a neighboring organoid (upper), and a CD8^+^ TEG killing a complete 13T organoid over 11 h (lower). Scale bars, 30 µm; time, h. **e**, Processed images from **d** showing 3D-rendered organoids (gray) at *t* = 0 and the CD4^+^ TEG or CD8^+^ TEG with their full track. Scale bars, 10 µm. **f**, UMAP embedding showing expression levels of NCAM1. Color gradient represents log_2_-transformed normalized counts of genes. **g**, Quantification of the percentage of dying 13T organoids (of total) after 10 h of coculture with either sorted NCAM1^–^CD8^+^ TEGs or NCAM1^+^CD8^+^ TEGs (*n* = 5 independent experiments, mean ± s.e.m.). Two-tailed unpaired *t*-test, *P* = 0.0001036. **h**, Schematic representation of fluorescent labelling strategy for CD8^+^ TEGs. **i**, Behavioral cluster difference (%) between NCAM1^–^CD8^+^ TEGs and NCAM1^+^CD8^+^TEGs cocultured with 13T organoids (*n* = 6 independent experiments, mean ± s.e.m.). Linear regression model fitting with each experimental replicate as a random effect: CL9, *P* = 0.0002 (***); CL8, *P* = 0.07 (·) ; CL2, *P* = 0.005 (**); CL1, *P* = 0.02 (*). **j**, Images of 13T organoids (gray) with NCAM^+^ super engager CD8^+^TEGs (top) and NCAM^–^ lazy and dying CD8^+^TEGs (bottom). Scale bars, 10 µm. **a**,**d**,**j**, Representative of *n* = 5, 3 and 5 independent experiments, respectively).

**Extended Data Fig. 4:**
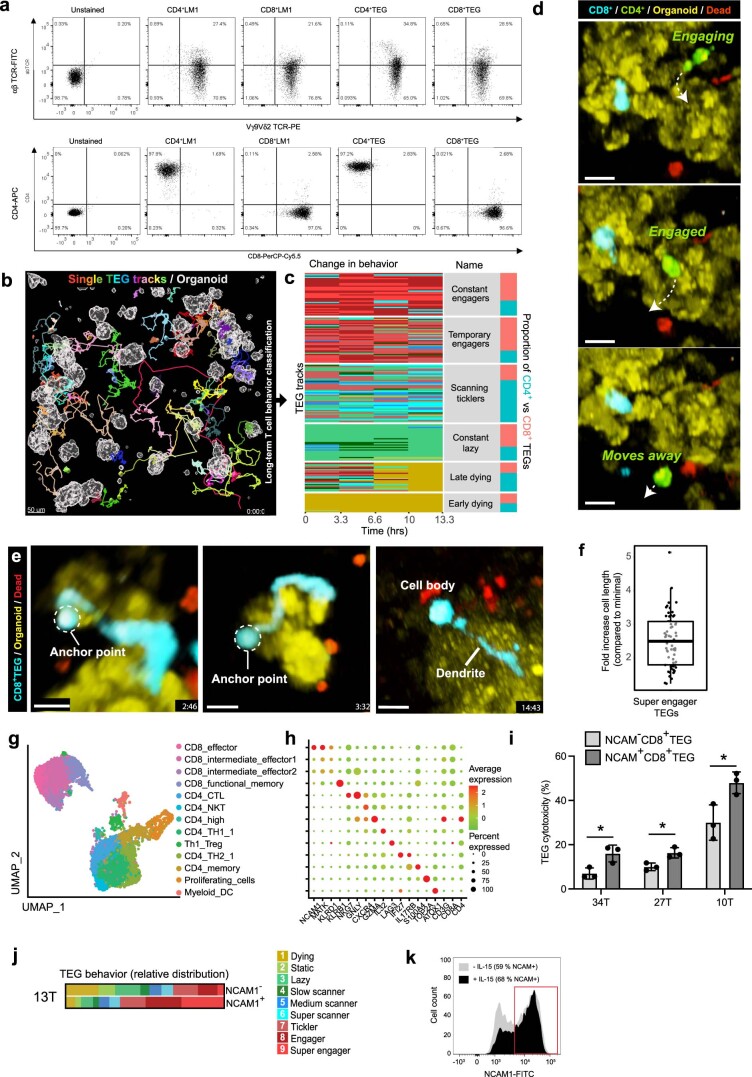
Unique targeting features of CD4^+^ and CD8^+^ TEGs and behavioral signatures in relation to NCAM1 expression. (**a**) Representative FACS plots showing CD4, CD8, αβ TCR and Vψ9/V82 TCR expression for cultured CD4^+^ and CD8^+^ LM1s or TEGs. (**b**) Representative image of long-term tracking of TEGs in co-culture with 13 T organoids (grey surface rendering at t = 0) showing full tracks (up to 20 hrs; rainbow-colored). Scale bar, 50 µm. (**c**) Time series color plot showing long-term tracks of TEGs (co-cultured with 13 T) and how they change their behavioral signature overtime for each time interval. (0- 3.3 hrs; 3.3-6.6 hrs; 6.6-10 hrs; 10-13.3 hrs). Colors indicate cluster identity for each TEG (see j). Tracks were classified into 6 different groups (named according to their most distinct behavior) and the proportion of CD4^+^TEG and CD8^+^TEG is indicated per group. TEGs were pooled from 3 independent experiments. (**d**) CD4^+^ TEG moving away from a 13 T organoid without killing. Scale bars, 20 µm. (**e**) Images showing 13 T organoids and CD8^+^ TEGs with defined anchor points. Scale bars, 10 µm. (**f**) Quantification of fold increase in cell length. Individual cells pooled from 6 independent experiments. Boxplot depicts the median, first and third quartiles, whiskers extend 1.5 times from the interquartile range. (**g)** UMAP plot shows distinct TEGs subsets unexposed to PDOs, pooled from three independent experiments. (**h**) Gene-expression dot plot of a curated set of differentially expressed genes in each cell subpopulation. Rows depict cell subpopulations as in g, while columns depict genes. (**i**) Quantification of breast cancer PDO targeting using a CellTiter-Glo® viability assay upon overnight co-culture with sorted NCAM1^−^CD8^+^TEGs or NCAM1^+^CD8^+^TEGs. Data corrected for organoid only responses. Unpaired T test: 34 T p = 0,0263; 27 T p = 0,0198; 10 T p = 0,0289. (n = 3 individual wells, representative data of 3 independent experiments; mean ± s.d.). (**j**) Relative behavioral cluster distribution of NCAM1^−^CD8^+^ TEGs or NCAM1^+^CD8^+^ TEGs co-cultured with 13 T PDOs. (**k**) FACS histogram plots showing NCAM1 expression in TEGs that were cultured in the absence (grey) or presence of IL-15 (black) for 10 days. (Representative data of 3 (b,d,e,k) or 4 (a) independent experiments).

**Extended Data Fig. 5:**
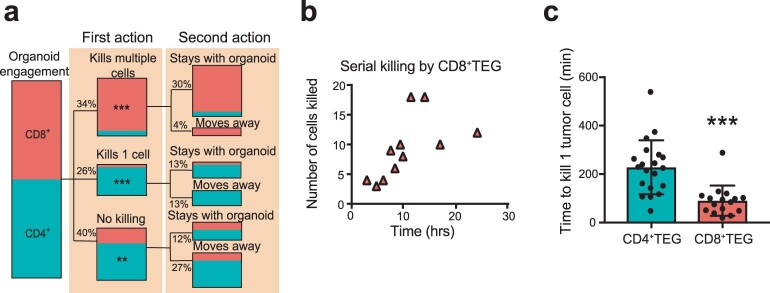
Analysis of TEG behavior and killing properties. (**a**) Quantification of the first action and second action of CD4^+^ and CD8^+^ TEGs after they engaged with an organoid. (n = 3 independent experiments). Hypergeometric test was used to analyze cell type enrichment in each category. ‘Kills multiple cells’ p < 0.0001; ‘Kills one cell’ p = 0.000015; ‘No killing’ p= 0.0018. (**b**) Quantification of the number of cells killed in a sequence by CD8^+^TEGs in time. (n = 3 independent experiments). (**c**) Quantification of the time it takes to kill one 13 T tumor cell for CD4^+^ TEGs and CD8^+^ TEGs (n = 3 independent experiments).

To link tumor-targeting behavior to population phenotypes, we next differentially labelled CD4^+^ and CD8^+^ TEGs in BC PDO cocultures (Fig. 4a and Extended Data Fig. 4a). This revealed that prolonged organoid contact and super engager behavior was a preferred feature of CD8^+^ TEGs, whereas CD4^+^ TEGs showed a higher proportion of lazy cells, slow scanners, medium scanners, super scanners and ticklers (Fig. 4a–c) characteristic of high movement and short organoid contact (Fig. 2c). Furthermore, long-term behavior classification and back-projection of cells classified in the live-tracked imaging dataset (Extended Data Fig. 4b,c) showed that single CD8^+^ TEGs, once engaged with an organoid, most often killed multiple cells consecutively (serial killing) (Fig. 4d,e, Extended Data Fig. 5a,b and Supplementary Video 1), a preferred feature of engineered T cells^31–33^. By contrast, CD4^+^ TEGs often moved away after organoid engagement without killing but occasionally targeted individual cells in different organoids (Fig. 4d,e and Extended Data Figs. 4d and 5a), thereby showing slower killing rates (Extended Data Fig. 5c). Thus, compared with CD4^+^ TEGs, CD8^+^ TEGs were shown to be more potent tumor-targeting cells with profound serial killing capacity. Serial killing by super engager CD8^+^ TEGs was characterized by attachment to PDOs using a defined anchor point from which surrounding cells were killed via long protrusions, intercalating between epithelial cells and extending their initial size up to fivefold (Fig. 4d and Extended Data Fig. 4e,f). We confirmed morphological plasticity and serial killing potential also for WT1 T cells and ROR1 CAR T cells analyzed through BEHAV3D (Fig. 3c,d and Supplementary Video 1). Remarkably, single CD8^+^ TEGs were able to kill entire organoids (up to 18 cells in 11 h; Fig. 4d, Extended Data Fig. 5b and Supplementary Video 1). This extent of serial killing and morphological plasticity of super engager CD8^+^ TEGs was uniquely revealed by the high spatiotemporal resolution character of BEHAV3D.

### Neural cell adhesion molecule 1 is associated with super engager behavior

Through single-cell RNA sequencing (scRNA-seq), we observed differential expression of neural cell adhesion molecule 1 (NCAM1) in CD8^+^ TEGs (Fig. 4f, Extended Data Fig. 4g,h and Supplementary Table 3). Although linked to cytotoxicity in both αβ and γδ T cells^34^, this surface marker has not been examined in the context of cellular immunotherapy. Making use of this differential NCAM1 expression, we provided proof of concept for engineered T cell functional selection by showing that sorted NCAM1^+^CD8^+^ TEGs have a greater capacity to kill various BC PDOs compared with NCAM1^−^CD8^+^ TEGs (Fig. 4g and Extended Data Fig. 4i). To identify behavioral mechanisms underlying this high killing potential, we prelabelled CD8^+^ TEGs with NCAM1 nanobodies (Fig. 4h) for direct comparison of NCAM1-positive and -negative populations within the same environment. NCAM1^+^CD8^+^ TEGs showed reduced dying and static behavior (Fig. 4i,j and Extended Data Fig. 4j), supporting a higher in vitro persistence. Strikingly, NCAM1^+^CD8^+^ TEGs additionally showed a significant increase in super engager behavior compared with NCAM1^–^CD8^+^ TEGs (Fig. 4i,j). Thus, surface marker expression can be linked to engineered T cell behavior, offering the opportunity to enrich for potent effector behaviors through cell selection. Functional skewing during engineered T cell production might also be feasible, because TEGs expanded in the presence of IL-15 expressed higher levels of NCAM1 (Extended Data Fig. 4k), in line with a role for IL-15 in NCAM1 induction^34^.

### Behavioral-transcriptomic profiling of TEGs

**Fig. 5:**
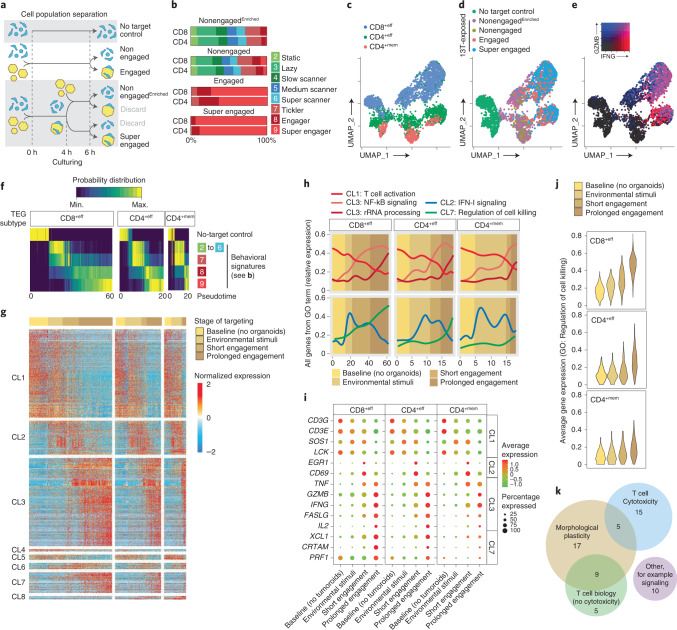
Behavioral-transcriptomic profiling of TEGs following PDO exposure, engagement and killing. **a**, Schematic representation of cell population separation for isolation and sequencing of super engaged, engaged, nonengaged, nonengaged^Enriched^ and no-target control TEGs. **b**, Distribution of the nine behavioral signatures described in Fig. 2b,c of the indicated behavior-enriched TEG populations isolated after 6 h of coculture with 13T PDOs. *n* = 6 independent experiments. **c**–**e**, UMAP embedding of pooled scRNA-seq profiles showing distribution of CD8^+eff^, CD4^+eff^ and CD4^+mem^ TEGs (**c**), the five behavior-enriched TEG populations described in **a** (**d**) and normalized gene expression of IFNG and GZMB (**e**). Colors represent log_2_-transformed normalized counts of genes. **f**, Heatmap representing the probability distribution of different behavioral signatures and no-target control over pseudotime for CD8^+eff^, CD4^+eff^ and CD4^+mem^ TEGs. Colors represent the scaled probability for each behavioral group. **g**, Heatmap showing normalized gene expression dynamics of TEGs following exposure to and engagement with 13T PDOs. Columns represent T cells ordered in pseudotime, rows represent gene expression grouped based on similarity, resulting in eight gene clusters. CLs 1–3 represent gene expression patterns shared among TEG subsets; CLs 4–8 show different expression dynamics between TEG subsets. Horizontal color bar (top) represents the corresponding stage of targeting based on data in **f**. **h**, Averaged gene expression over pseudotime for all genes from indicated GO terms for the indicated TEG subtypes. Background color shading represents the corresponding stage of targeting; line colors indicate GO terms. **i**, Gene expression dot plot for a curated subset of genes at different stages of targeting. Rows depict genes, dot color gradient indicates average expression while dot size reflects the proportion of cells expressing a particular gene (%). **j**, Violin plots for different TEG subtypes showing averaged expression of genes related to GO term ‘Regulation of cell killing’ enriched in CL7 from **g**. Colors indicate different stages of targeting. **k**, Venn diagram depicting common and unique functions from 61 conserved genes comprising a (serial) killer gene signature. **b**–**d**, T cells pooled from two independent experiments).

**Extended Data Fig. 6:**
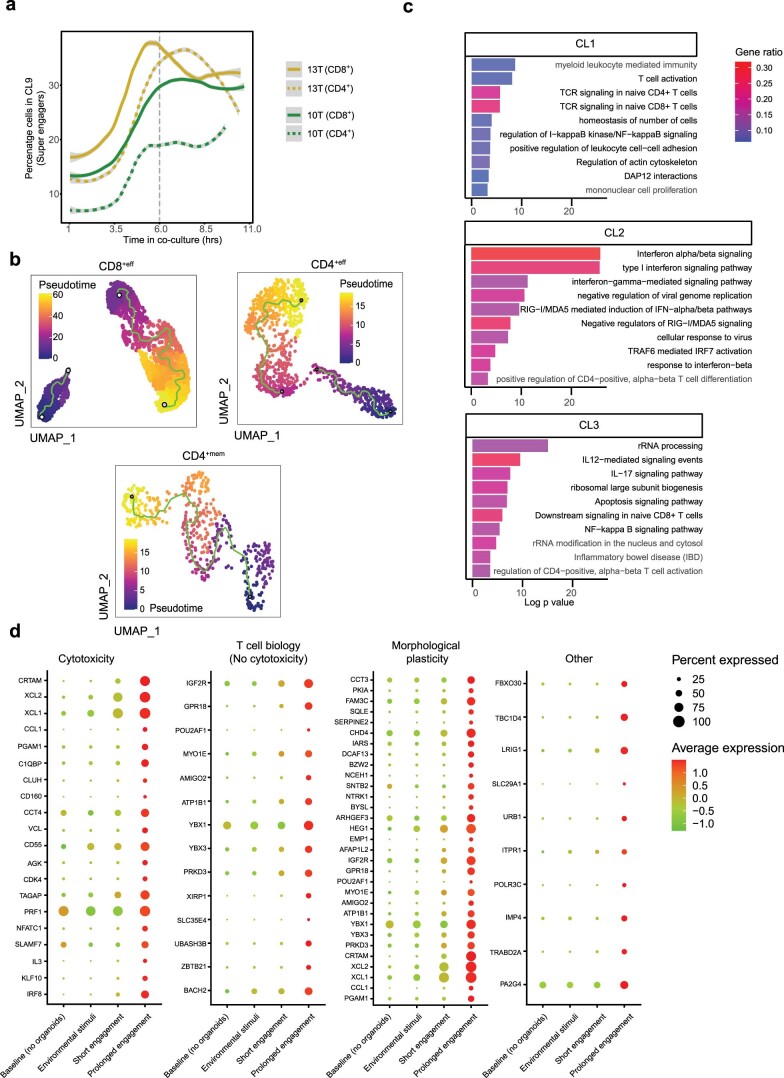
Behavior-guided transcriptomics of TEGs co-cultured with 13T organoids. (**a**) Dynamic change of the percentage of TEGs exhibiting *super engager* behavior (C9) over time in co-culture. Color denotes TEGs co-cultured with 13 T or 10 T organoids and line type CD4^+^ (dashed) or CD8^+^ (solid) TEGs. The 6 hrs time point was selected for single cell TEG sequencing (dashed grey line). (**b**) Separate UMAP embeddings showing inferred pseudo-time trajectory of CD8^+eff^, CD4^+eff^ and CD4^+mem^ TEGs. Color scale represents the inferred pseudotime. (**c**) Functional enrichment analysis for biological processes and pathways from gene clusters (CL) that are downregulated (CL1), upregulated (CL3) or transiently expressed (CL2) over the pseudotime trajectory of TEGs targeting 13 T organoids. CL1-3 are represented in Fig. 5g. (**d**) Gene- expression dot plot of the 61 conserved genes composing the (serial) killer gene signature separated by function. Rows depict genes, while columns depict stage of targeting. Dot color gradient indicates average expression, while size reflects the proportion of cells expressing a particular gene.

To generate insight into the transcriptional programs that underlie tumor-targeting dynamics revealed by BEHAV3D, we next performed single-cell transcriptomic profiling of TEG populations enriched for different behavioral signatures following coculture with BC PDOs, including a TEG population containing >90% super engagers (Fig. 5a,b, Extended Data Fig. 6a and Supplementary Video 1). For each main TEG subset identified, effector CD8^+^ (CD8^+eff^), effector CD4^+^ (CD4^+eff^) and memory CD4^+^ (CD4^+mem^), profound transcriptional changes were observed following 6 h of coculture with highly targeted 13T organoids as compared with baseline (no-target control) (Fig. 5c–e), showing that dynamic interplay with PDOs shapes the TEG transcriptomic profile. We developed a method of behavioral probability mapping inferred from pseudotemporal ordering (Extended Data Fig. 6b) of the sequenced TEG populations (Fig. 5f), allowing us to pinpoint gene programs in TEGs regulated by environmental stimuli, short PDO engagement and prolonged PDO engagement (Fig. 5f,g).

This revealed dynamic transcriptional programs highly conserved between CD8^+eff^, CD4^+eff^ and CD4^+mem^ TEGs (Fig. 5g; gene clusters 1–3, 85% of genes; Supplementary Table 4). These programs included genes either downregulated (CL1) or upregulated (CL3) by environmental stimuli or engagement with PDOs, as well as those transiently expressed (CL2) along the pseudotime trajectory (Fig. 5g; GO terms per cluster shown in Extended Data Fig. 6c). This differential dynamic expression matched with known gene function, confirming robust ordering of TEGs as shown by genes related to the CD3 signaling complex (*LCK*, *SOS1*, *CD3E*, *CD3G*, *CL1*; GO term ‘T cell activation’) known to be downregulated following T cell activation^35^ in CL1 (Fig. 5h). NF-kB signaling, critical for tumor control^36^, and effector molecules, including FASLG, IFNG, GZMB and TNF, were found in CL3, with NF-kB signaling induced by environmental stimuli reaching maximum expression following prolonged PDO engagement, while effector molecules appeared upon engagement (Fig. 5i). In addition, CL3 contained genes related to ribosomal RNA processing that increased only following prolonged engagement with organoids (Fig. 5h), consistent with accelerated protein production in T cells following TCR engagement^37,38^. Finally, CL2 contained the early activation markers CD69 and EGR1 with peak expression following short organoid engagement, in line with IL-2 (CL3), known to be induced by EGR1 (ref. ^39^), upregulated towards the end of the trajectory (Fig. 5i). Thus, through our behavior-guided transcriptomics approach we robustly identified dynamic gene orchestration of TEGs during tumor targeting.

### Gene signature related to (serial) killing super engager TEGs

Of those gene sets regulated in a TEG subset-specific manner (CL4–8, 15% of genes), CL7 contained genes mainly induced following prolonged organoid engagement, including cytotoxic genes (for example, *PRF1*, *CRTAM*, *XCL1*; (Fig. 5h,i; GO: Regulation of cell killing). This cluster of genes was specifically induced in super engager CD8^+eff^ and, to a lesser extent, in CD4^+eff^ TEGs but was almost absent in CD4^+mem^ TEGs (Fig. 5j), associating this gene cluster with potent (serial) killing T cells (Fig. 4d–g). Analysis of TEGs derived from a different donor and cocultured with another BC PDO (10T) confirmed that 61 out of the 83 genes of CL7 represent a conserved ‘killer’ gene signature (Supplementary Table 5). Of these, we identified 20 genes related to T cell activation and cytotoxicity and 14 related to other T cell functions (Fig. 5k and Extended Data Fig. 6d). However, we found 27 genes with no previously described T cell function (Fig. 5k and Extended Data Fig. 6d). Overall, half of all conserved signature genes (31/61) and 17 out of the 27 genes were related to morphological plasticity processes including motility, cytoskeleton remodeling and adhesion (Extended Data Fig. 6d). Given that morphological plasticity is a key determinant of cell migration, many of the identified genes were found to have a role in promotion of tumor cell migration and invasion, including ECM production and mesenchymal state induction (*HEG1*, *BZW2*, *DCAF13*, *SQLE*, *PKIA*). For some of these genes, such as *CCT3* or *AFAP1L2*, the mechanism promoting migration is yet to be described. In line with the prolonged organoid engagement behavioral feature of super engager TEGs (Fig. 2c), we also found various genes related to cell adhesion including *NCEH1*, *BYSL* and *EMP1*. Finally, some genes had an additional function related to neurite outgrowth and dendritic pruning (*SERPINE2*, *CHD4*, *NRTK1*), potentially matching the long protrusion that was observed to occur in these serial killing TEGs (Fig. 4e, Extended Data Fig. 4e,f and Supplementary Video 1). Thus, the behavioral-transcriptomics module of BEHAV3D identified a specific gene signature induced in (serial) killing super engager TEGs.

**Extended Data Fig. 7:**
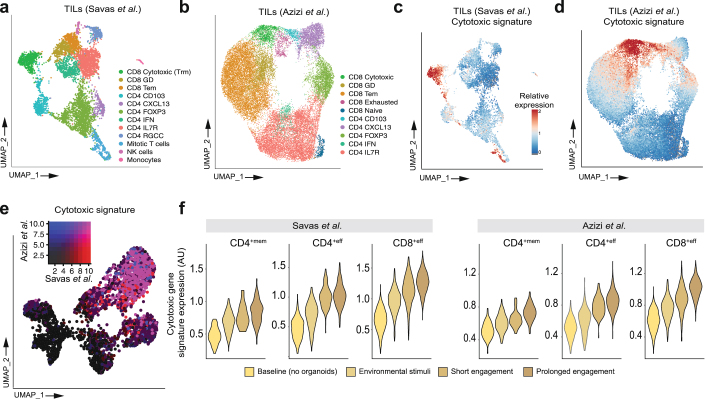
Overlap between cytotoxic signature of tumor-infiltrating T cells and *super engager* TEG gene expression. (**a,b**) UMAP embedding color-coded for different populations of tumor-infiltrating lymphocytes (TILs) isolated from human breast cancer tumor samples from the Savas *et al*.^41^ (a; 310 genes) or Azizi *et al*.^40^ (b; 543 genes) study. (**c,d**) UMAP embedding of TILs showing relative expression of a cytotoxic gene signature identified in the Savas *et al*. (c) or Azizi *et al*. (d) dataset. (**e**) UMAP embedding of TEGs enriched for different behaviors (see Fig. 5c-e) showing normalized gene expression projection of the Savas *et al*. or Azizi *et al*. cytotoxic signature. Colors represent the log^2^ transformed normalized counts of genes. (**f**) Violin plots for different TEG subtypes showing expression of the cytotoxic gene signature identified in Savas *et al*. (left panel) or Azizi *et al*. (right panel). Colors indicate different stages of targeting.

To provide some context as to how induced gene signatures in TEGs relate to in vivo tumor targeting, we compared our behavior-guided transcriptomics results with two published scRNA-seq datasets on tumor-infiltrating lymphocytes (TILs) obtained from patients with breast cancer^40,41^. Both datasets identified a potent CD8^+^ T cell population defined by a cytotoxic gene signature (310 (ref. ^41^) and 533 genes^40^) and prognostic value for patient survival (Extended Data Fig. 7a–d and Supplementary Table 6). When comparing the gene profiles of these in-vivo-identified cytotoxic CD8^+^ TILs with our data, we observed a substantial overlap with the gene signature of CD8^+^ TEGs that were selected based on super engager behavior (Extended Data Fig. 7e). The highest relative expression of the cytotoxic gene signatures from both datasets above^41,40^ was observed in CD8^+eff^ TEGs after prolonged organoid engagement (Extended Data Fig. 7f). These data thereby demonstrate that the gene signatures related to potent tumor targeting in vivo of patients with breast cancer overlap with that of super engager TEGs, supporting the in vivo relevance of our approach.

### PDOs shape the dynamic gene signature of TEG during tumor targeting

**Fig. 6:**
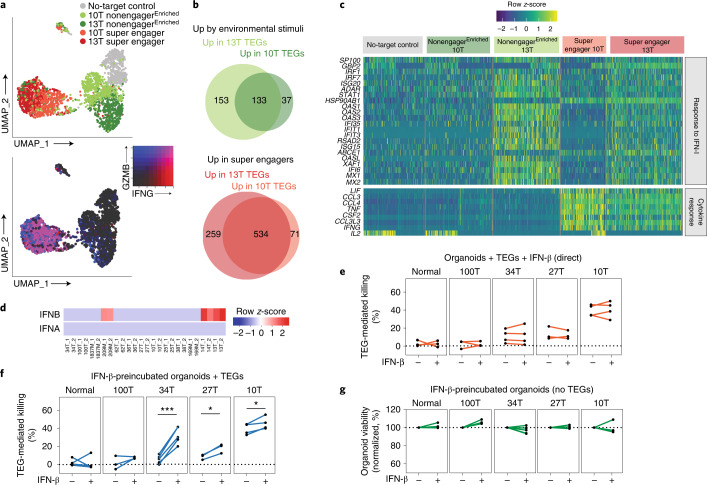
IFN-I signaling in PDOs primes TEG efficacy. **a**, Top: UMAP embedding of pooled scRNA-seq profiles from super engaged and nonengaged^Enriched^ TEG populations cocultured with either 13T or 10T PDOs, and from no-target control T cells. TEGs are colored according to experimental condition. Bottom: UMAP plot showing expression levels of *IFNG* and *GZMB*. Colors represent log_2_-transformed normalized counts of genes. **b**, Venn diagrams depicting common and unique genes upregulated (up) in TEGs following 13T and 10T organoid exposure (top, environmental stimuli) or prolonged engagement (bottom, super engagers). **c**, Heatmap of gene expression for genes involved in functional annotations of interest in response to IFN-I, cytokine response), grouped according to TEG populations. **d**, *IFNA* and *IFNB* expression in PDOs from the BC panel in Fig. 1d. 1 and 2 indicate different experimental replicates. **e**–**g**, Quantification of dying single organoids in the presence or absence of recombinant IFN-β for the following conditions: organoids cocultured with TEGs with direct addition of IFN-β, corrected for responses of LM1 control T cells (**e**); organoids preincubated with IFN-β for 24 h before coculture with TEGs, corrected for responses of LM1 control T cells (**f**); and organoids preincubated with IFN-β for 24 h and cultured in the absence of TEGs (**g**). Lines connect experimental replicates. **f**, Statistical analysis was performed by paired *t*-test: 34T IFN-β versus 34T control, *P* < 0.0006 (***); 27T IFN-β versus 27T control, *P* < 0.0216 (*); 10T IFN-β versus 10T control, *P* < 0.0402 (*). See Supplementary Table 8 for summary of replicates in **e**–**g**.

**Extended Data Fig. 8:**
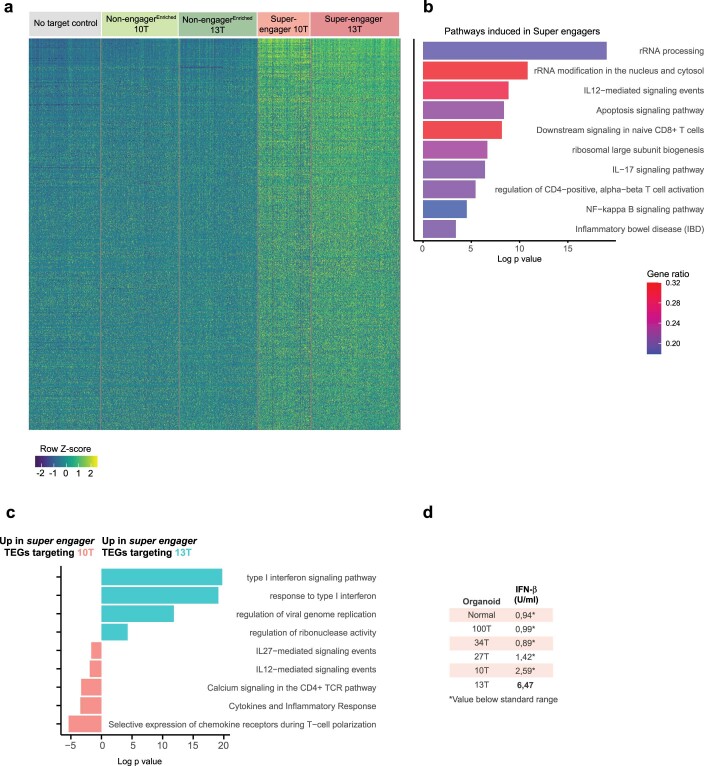
Behavior-guided transcriptomics of TEGs co-cultured with 13T and 10T organoids. (**a**) Heatmap showing normalized gene expression of behavior-enriched TEG populations co-cultured with 10 T or 13 T organoids, or cultured without PDOs (*No target control*). Columns represent cells ordered by TEG populations and rows represent the expression of genes. Shown are 534 genes induced upon prolonged organoid engagement (*super engagers*) in both 10 T and 13 T co- cultures from Fig. 6b. (**b**) Functional enrichment analysis (conserved biological processes and pathways) of genes induced in both 10T- and 13T-co-cultured *super engager* TEGs (shown in A). (**c**) Functional enrichment analysis of genes differentially expressed between 10T- and 13T-co- cultured *super engager* TEGs. Top differentially regulated biological processes and pathways are shown. (**d**) IFN-ß concentration measured for the different organoid cultures in Fig. 6e-g.

To further explore our behaviorally guided transcriptomics approach, we next compared behavior-enriched TEG populations cocultured with either highly sensitive (13T) or intermediately targeted (10T) BC PDOs. Distinct uniform manifold approximation and projection (UMAP) embedding of different TEG populations (Fig. 6a) indicated that patient-specific organoid exposure influences the dynamic TEG transcriptional profile—41 and 61%, respectively, of upregulated genes by environmental stimuli or following prolonged PDO engagement in super engagers were common between 10T- and 13T-cocultured TEGs (Fig. 6b, Extended Data Fig. 8a,b and Supplementary Table 7). Common super-engager-related gene signatures included rRNA processing, NF-kB signaling and cytokine signaling (Extended Data Fig. 8b), and matched CL3 gene signatures (Extended Data Fig. 6c). However, 10T-cocultured TEGs were characterized by induction of high cytokine expression following prolonged PDO engagement, including *TNF*, *IFNG* and *IL2*, whereas IFN-I signaling genes were uniquely induced in TEGs cocultured with highly sensitive 13T (Fig. 6c and Extended Data Fig. 8c).

### IFN-β primes PDOs for TEG-mediated killing

IFN-I signaling plays fundamental roles in antitumor immunity, but with diverse and sometimes opposing functions reported for both tumor and immune cells, thereby making it difficult to fully comprehend and therapeutically exploit these effects^42^. IFN-I signaling was detected in 13T BC PDOs (Extended Data Fig. 2c), which most prominently showed increased RNA levels of the upstream mediator IFN-β, but not IFN-α, among our collection of PDOs (Fig. 6d). Secretion of IFN-β was confirmed by Luminex (Extended Data Fig. 8d), implying that IFN-β was the main mediator of IFN-I signaling observed in 13T. Interestingly, peak induction of IFN-I signaling in 13T-cocultured TEGs was detected in nonorganoid-engaging TEGs (from static to super scanner behavior), in line with a secreted source of IFN-β, while the pathway was shut down in super engager TEGs, suggesting a limited role of IFN-I signaling in direct killing behavior (Fig. 5f–h). The addition of recombinant IFN-β to cocultures of TEGs with various BC PDOs showing low to medium sensitivity to TEG therapy (100T, 34T, 27T and 10T) indeed did not affect TEG targeting efficacy (Fig. 6e). However, 34T, 27T and 10T organoids pretreated with IFN-β showed increased TEG-mediated killing while IFN-β treatment did not impact organoid viability by itself (Fig. 6f,g). These data support the premise that IFN-β has limited impact on the killing capacity of super engager TEGs, confirming that dynamic IFN-I signaling is mainly associated with static to scanner behavior. However, IFN-I signaling increases the sensitivity of BC PDOs to TEG therapy. Thus, behavior-guided TEG transcriptomics in relation to the type of organoid exposure shows that IFN-β primes PDOs for targeting by TEGs. This illustrates the potential of BEHAV3D to improve understanding and guide combinatory treatment approaches in a patient-specific manner.

## Discussion

Here we provide an organoid-based, 3D imaging-transcriptomic platform, BEHAV3D, for understanding the mode of action of cellular anticancer immunotherapies in a patient-specific manner and apply it to diverse solid-tumor PDO models and multiple engineered T cell products. With BEHAV3D we have demonstrated differences in behavior between various engineered T cell products, uncovered the gene signature associated with serial killing, designed an optimal sequence of T cell combination therapy and provided proof of concept for a cell selection strategy to enrich for potent tumor-targeting behavior (Supplementary Discussion). Thus, BEHAV3D integrates multiple single-cell readouts (Supplementary Video 1) to offer a comprehensive platform with potential for broadening the implementation of cellular immunotherapy for solid tumors.

## Methods

### Human material

All human BC and head and neck PDO samples were retrieved from a biobank through the Hubrecht Organoid Technology (HUB; www.hub4organoids.nl). Authorizations were obtained by the medical ethical committee and biobank research ethics committee of UMC Utrecht (UMCU) at the request of HUB, to ensure compliance with the Dutch Medical Research Involving Human Subjects Act. Normal breast organoids were generated from milk obtained via the Moedermelkbank Amsterdam (Amsterdam UMC). Primary patient-derived DMG cultures (no. DMG-VI/SU-DIPG-VI) were kindly provided by M. Monje (Stanford University), M. Vinci (Ospedale Pediatrico Bambino Gecù, nos. DMG-002/OPBG-DIPG-002 and DMG-004/OPBG-DIPG-004-aa) and A. M. Carcaboso (Hospital San Juan de Dios, no. DMG-007/HSJD-DIPG-007). For TEG and WT1 T cell generation, peripheral blood of anonymous healthy donors was purchased from the Dutch blood bank (Sanquin). For CAR T cell generation, cord blood was collected with approval from the Ethical Committee of UMCU. Informed consent was obtained from all donors.

### Animal material

NOD.Cg-PrkdcscidIl2rgtm1Wjl/SzJ (NSG) mice were purchased from Charles River Laboratories. Experiments were conducted with permission from the Animal Welfare Body Utrecht (nos. 4288-1-08 and 4288-1-09) as per current Dutch laws on animal experimentation. Mice were housed under 45–65% humidity and a daily 12/12-h light/dark regime, in sterile conditions using an individually ventilated cage system and fed with sterile food and water. Irradiated mice were given sterile water with antibiotic ciproxin for the duration of the experiment. Mice were randomized with equal distribution by age and initial weight measured on day 0 and divided into groups of ten (13T) or 15 (169M).

### Organoid culture

Breast cancer and normal breast organoids were seeded in basement membrane extract (BME, Cultrex) in uncoated 12-well plates (Greiner Bio-one) and cultured as described previously^29,43^. Briefly, Advanced DMEM/F12 was supplemented with penicillin/streptomycin (pen/strep), 10 mM HEPES, GlutaMAX (adDMEM/F12+++), 1× B27 (all Thermo Fisher), 1.25 mM *N*-acetyl-l-cysteine (Sigma-Aldrich), 10 mM nicotinamide (Sigma-Aldrich), 5 μM Y-27632 (Abmole), 5 nM Heregulin β-1 (Peprotech), 500 nM A83-01 (Tocris), 5 ng ml^–1^ epidermal growth factor (Peprotech), 20 ng ml^–1^ human fibroblast growth factor (FGF)-10 (Peprotech), 10% Noggin-conditioned medium^20^, 10% Rspo1-conditioned medium^44^ and 0.1 mg ml^–1^ primocin (Thermo Fisher); and, in addition, with 1 μM SB202190 (Sigma-Aldrich) and 5 ng ml^–1^ FGF-7 (Peprotech) for PDO propagation (type 1 culture medium^43^), or with 20% Wnt3a-conditioned medium^44^, 0.5 μg ml^–1^ hydrocortisone (Sigma-Aldrich), 100 μM β-estradiol (Sigma-Aldrich) and 10 mM forskolin (Sigma-Aldrich) for normal organoid propagation (type 2 culture medium^43^). Organoids from passages 5–30 after cell isolation were used for T cell coculture.

For T cell coculture, organoids were recovered from the BME by resuspension in TrypLE Express and collected in adDMEM/F12+++ (BC and head and neck cancer PDOs) or resuspended and collected in adDMEM/F12+++ (DMG PDOs). Organoid suspensions were filtered through a 70-μm strainer (Greiner) to remove large organoids and pelleted before coculture.

### T cells engineered to express a γδ TCR (TEGs and LM1s)

TEG001 (T cells engineered to express a highly tumor-reactive Vγ9Vδ2 TCR)^6,45,46^, LM1s (mock T cells engineered to express a mutant Vγ9/Vδ2 TCR with abrogated function)^8^ and TEG011 (mock T cells engineered to express HLA-A*24:02-restricted Vγ5/Vδ1 TCR, used as control for in vivo studies)^47,48^ were produced as previously described^8^. Briefly, packaging cells (Phoenix-Ampho) were transfected with helper constructs gag-pol (pHIT60), env (pCOLT-GALV) and pMP71 retroviral vectors containing both Vγ9/Vδ2 TCR chains separated by a ribosomal-skipping T2A sequence, using FugeneHD reagent (Promega). Human peripheral blood mononuclear cells (PBMCs) from healthy donors were preactivated with anti-CD3 (30 ng ml^–1^; Orthoclone OKT3, Janssen-Cilag) and IL-2 (50 IU ml^–1^; Proleukin, Novartis) and subsequently transduced twice with viral supernatant within 48 h in the presence of 50 IU ml^–1^ IL-2 and 6 mg ml^–1^ polybrene (Sigma-Aldrich). TCR-transduced T cells were expanded by stimulation with anti-CD3/CD28 Dynabeads (500,000 beads 10^–6^ cells; Life Technologies) and IL-2 (50 IU ml^–1^). Thereafter, TCR-transduced T cells were depleted of nonengineered T cells by magnetic-activated cell sorting (MACS) as previously described^8^. This depletion protocol establishes a predominantly αβ TCR^–^ population (Extended Data Fig. 4a), which has been shown to result in complete loss of alloreactivity (Extended Data Fig. 1e)^45^. To separate CD4^+^ and CD8^+^ TEGs and LM1s, we performed positive selection using either CD4 or CD8 Microbeads (Miltenyi Biotech) following the manufacturer’s instructions. After incubation with magnetic microbeads, cells were applied to LS columns and CD4^+^ or CD8^+^ TEGs or LM1s were selected by MACS. After the MACS selection procedure, Vγ9/Vδ2 TCR^+^ CD4^+^ or Vγ9/Vδ2 TCR^+^ CD8^+^ subsets of TEGs were stimulated every 2 weeks using a rapid expansion protocol^8^ where TEGs were cultured in ‘T cell culture medium’ (RPMI-GlutaMAX supplemented with 2.5–10% human serum (Sanquin), 1% pen/strep and 0.5 M beta-2-mercaptoethanol) on a feeder cell mixture comprising sublethally irradiated allogenic PBMCs, Daudi and LCL-TM in the presence of IL-2 (50 U ml^–1^), IL-15 (5 ng ml^–1^; both R&D Systems) and PHA-L (1 μg ml^–1^; Sigma-Aldrich). To monitor the purity of CD4^+^ and CD8^+^ TEGs, as well as the absence of allogenic irradiated feeder PBMCs, cells were analyzed weekly by flow cytometry before functional assays using the antibodies anti-pan γδTCR-PE (Beckman Coulter), anti-αβTCR-FITC (eBioscience) anti-CD8-PerCP-Cy5.5 (Biolegend) and anti-CD4-APC (Biolegend). TEGs of purity <90% were reselected as described above. TEGs were used for coculture assays 4–5 days after the last IL2/IL15/PHA-L stimulation.

### Live-cell imaging of T cells and organoid cocultures

Engineered T cells (20,000) were cocultured with normal organoids, PDOs or control cell lines (Daudi or HL-60) in an effector/tumor cell (E:T) ratio of 1:30 or 1:25 (only for Fig. 4d,e and Extended Data Fig. 5a). CD4^+^ and CD8^+^ TEGs were mixed in a 1:1 ratio immediately before plating. Cells were incubated in 96-well, glass-bottom SensoPlates (Greiner) in 200 µl of ‘coculture medium’: 50% type 1 organoid culture medium, 50% ‘TEG assay medium’ (RPMI-GlutaMAX supplemented with 10% fetal calf serum and 1% pen/strep), 2.5% BME and pamidronate for the accumulation of the phosphoantigen IPP to stimulate tumor cell recognition^8^ (1:2,000). Coculture medium was supplemented with both NucRed Dead 647 (two drops ml^–1^; Thermo Fisher) and TO-PRO-3 (1:3,000; Thermo Fisher) for fluorescent labelling of living and dead cells (‘Imaging medium’). The combination of NucRed Dead 647 and TO-PRO-3 labels dead cells when excited with a 633-nm laser and living cells with a 561-nm laser (Extended Data Fig. 1a,b). Both were combined to achieve the optimal fluorescent intensity ratio between dead and living cells for live-cell imaging. Before coculture, TEGs were incubated with eBioscience Cell Proliferation Dye eFluor 450 (referred to as eFluor-450; 1:4,000; Thermo Fisher) in PBS for 10 min at 37 °C to fluorescently label all T cells. When CD4^+^ and CD8^+^TEGs were simultaneously imaged, both eFluor-450 and Calcein AM (1:4,000; Thermo Fisher) were used to label the different TEG subsets in PBS for 10 min at 37 °C. For NCAM1 prelabelling experiments, a combination of eFluor-450 (1:4,000; Thermo Fisher) and Hilyte-488-conjugated NCAM1 nanobodies (1:400; QVQ) was used to label CD8^+^ TEGs in PBS for 20 min at 37 °C before coculture. The plate was placed in a LSM880 (Zeiss Zen Black Edition v.2.3) microscope containing an incubation chamber (37 °C, 5% CO_2_) and incubated for 30 min to ensure settling of TEGs and organoids at the bottom of the well. The plate was imaged for up to 24 h with a Plan-Apochromat ×20/0.8 numerical aperture dry objective with the following settings: online fingerprinting mode, bidirectional scanning, optimal Z-stack step size, Z-stack of 60 μm in total and time series with either a 30-min interval (up to 60 conditions simultaneously; resolution 512 × 512) or a 2-min interval (up to four or ten conditions simultaneously; resolution 512 × 512 and 200 × 200, respectively). To minimize photobleaching of NCAM1-prelabelled TEGs, the 488-nm laser was activated during only one Z-stack each hour within the first few hours of imaging. Directly after imaging, production of IFN-γ in the supernatant was quantitated using an ELISA-ready-go! Kit (eBioscience) and cell pellets were used to measure organoid viability with the CellTiter-Glo Luminescent Cell Viability Assay (Promega).

### IFN-β stimulations

PDOs were harvested as described above and incubated in 96-well, round-bottom culture plates (Thermo Fisher) in 100 µl of type 1 organoid culture medium, supplemented with 2.5% BME and with or without the presence of 100 pg ml^–1^ recombinant human IFN-β (Peprotech). After 24 h of incubation (37 °C, 5% CO_2_), TEGs or LM1s were added to either IFN-β-preincubated or unstimulated organoids (E:T ratio 1:30) in 100 µl of TEG assay medium, supplemented with 2.5% BME and pamidronate (1:1,000) and with or without the presence of 100 pg ml^–1^ recombinant human IFN-β (Peprotech). Medium without T cells was added for ‘organoid only’ controls. After 16 h of incubation (37 °C, 5% CO_2_), plates were used to measure organoid viability using the CellTiter-Glo Luminescent Cell Viability Assay.

### In vivo targeting by TEGs

Adult female NSG mice (15–16 weeks old) received sublethal total body irradiation (1.75 Gy) and subcutaneous implantation of a β-estradiol pellet (Innovative Research of America) on day –1. On day 0, PDOs (1 × 10^6^ 13T or 0.5 × 10^6^ 169M organoid cells in 100 μl of BME per mouse) were prepared as described previously^43^ for subcutaneous injection in the right flank on day 0, and mice received two injections of 10^7^ TEGs or TEG011 mock cells on days 1 and 6 in pamidronate (10 mg kg^–1^ body weight) as previously reported^7^. On day 1, together with the first T cell injection, all mice also received 0.6 × 10^6^ IU of IL-2 (Proleukin, Novartis) in incomplete Freund’s adjuvant (IFA; MD Bioproducts) subcutaneously. Tumor volume was measured once per week using a digital caliper and calculated by the following formula: 0.4 × (length x width^2^). Mice were monitored at least twice per week for weight loss and clinical appearance scoring (scoring parameters included hunched appearance, activity, fur texture, piloerection and respiratory/breathing problem). Humane endpoint was reached either when mice experienced 20% weight loss from initial weight, tumor volume reached 2 cm^3^ or when a clinical appearance score of 2 was reached for an individual parameter or an overall score of 4. In no case was the tumor burden exceeded.

### Image processing

For 3D visualization, cell segmentation, extraction of statistics and time-lapse videos were processed with Imaris (Oxford Instruments) v.9.2–9.5. The Channel Arithmetics Xtension was used to create new channels for specific identification of organoids (live and dead) and eFluor-450-labelled or calcein AM-labelled T cells (live and dead) and to exclude cell debris. The Surface and ImarisTrack modules were used for object detection and automated tracking of both T cells (autoregressive motion) and organoids (‘connected components’ or no tracking). The Distance Transformation Xtension was used to measure the distance between TEGs and organoids, with thresholds for defining organoid–T cell interactions visually determined. For tracked TEGs, time-lapse data containing the coordinates of each cell, the values of cell speed, mean square displacement, distance to organoids and dead cell dye channel intensity were exported. For experiments with NCAM1 prelabelling, the mean intensities of the NCAM1 channel per T cell were exported. For tracked organoids, time-lapse data containing the coordinates of each organoid, the surface area, volume and mean dead cell dye channel intensity were exported.

### PDO killing dynamics

To quantify the cell death dynamics of PDO cultures, >5,000 single organoids were analyzed at each time point (48 in total). The mean dead cell dye intensity within single organoid surfaces was quantified and rescaled to a range between 0 and 100 per experiment to normalize for variation in absolute dead cell dye intensity. To analyze whether organoid sensitivity to TEGs was dependent on initial organoid size, we compared the initial area (0 h) of organoids killed by TEGs at 10 h compared with the area of TEGs remaining alive at 10 h.

### T cell dynamics analysis and multivariate time series clustering

For the analysis of TEG behavior over time, the following parameters were used: T cell death, contact with organoids, speed, square displacement and interaction with other T cells. For each T cell time series, linear interpolation was used to estimate the values in several cases of missing time points. To compare time series independently of their length, cell tracks were cut to a length of 3.3 h. Similarity between distinct cell tracks was measured using a strategy that allows for best alignment between time series, previously applied for mitotic kinetics^49^ or temporal module dynamics comparisons^50^. A cross-distance matrix based on multivariate time series data was computed using the dynamic time-warping algorithm. To visualize distinct cell behaviors in two dimensions, dimensionality reduction on the multidimensional feature count table was performed by the UMAP method^51,52^. Clustering was performed using the k-means clustering algorithm with outlier detection. To confirm the identity of each cluster, T cell cluster assignments were back-projected to visualize the surfaces and tracks of particular T cell populations in the imaging dataset (Fig. 2a and Extended Data Figs. 3a,b and 4b).

### Cell behavior classification using a random forest classifier

For standardized integration of new experiments, we used a random forest classification approach^53^ to relate cell behavior to the nine behavioral signatures that we found in our global TEG behavior atlas (Fig. 2b). To allow for inclusion of experiments with a low E:T ratio of 1:25, where the parameter of T cell interaction would be influenced as compared with the standard E:T ratio of 1:30, the following parameters were used: T cell death, organoid contact, speed and square displacement. The reference dataset used to build the global TEG behavior atlas was split into cell tracks for use as either a training dataset (95%) or a test dataset (5%). To reduce dimensionality, for each cell track four time series descriptive statistics were quantified and used to train the classifier. For numeric variables, the following measures were computed for each cell track: mean, median, the top 90% of the distribution and standard deviation. For binary values, such as contact with organoids, the mean was calculated as well as the mean and maximum of cumulative interaction. The random forest classifier was trained using 100 trees on the above-mentioned variables using the nine behavioral signatures as labels (Extended Data Fig. 3c,d). The test dataset was used to assess accuracy of the classifier and to determine in which behavioral signatures the errors occurred (Extended Data Fig. 3e). A slightly updated version of the classifier was used in Fig. 3.

### Correlation between TEG behavior and organoid killing dynamics

To estimate the correlation between onset of death in individual organoids and engagement with T cells belonging to the engaging clusters (CL7–9), we implemented a technique of sliding window correlation analysis previously used for functional brain connectivity^54^ and genome analysis^55^. We calculated the Pearson correlation coefficient between the cumulative number of organoid contacts with TEGs from each cluster and the increase in dead cell dye intensity in each over a sliding window of 3 h (Fig. 2f and Extended Data Fig. 3k).

### NCAM1 prelabelling quantification using 3D imaging data

Behavioral classification of NCAM1-prelabelled TEGs was performed as described above, by prediction of behavioral signatures with the random forest classifier. NCAM1^+/–^ TEGs were identified based on an NCAM1 intensity threshold in individual TEGs, visually defined at the time points where the 488-nm laser was turned on. To ensure inclusion of true NCAM1^–^ or NCAM1^+^ TEGs, two intensity thresholds were defined.

### Pseudotime trajectory inference

Two experimental SORT–seq replicates of TEGs cocultured with 13T PDOs, generated as described above, were used for trajectory interference (Extended Data Fig. 6b). Proliferating T cells were excluded from the analysis because they did not show any dynamic inflammatory genes during analysis. Afterwards, the gene expression table was log normalized with a 10,000 scaling factor. Shared nearest-neighbor, graph-based clustering was done as described above at a resolution of 2. Based on marker gene expression of CD8, CD4 and IL17RB^56^, TEGs were subclustered into three subtypes: IL17RB^−^CD8^+eff^, IL17RB^−^CD4^+eff^ and IL17RB^+^CD4^+mem^. Downstream analyses were performed on each subset separately and compared with each other where mentioned.The RunFastMNN function from the SeuratWrappers package was utilized to correct for batch effects between the two SORT–seq replicates. We used the package Monocle3 (ref.^57^) to infer the pseudotime trajectory and significantly dynamic genes for each T cell subtype. For each cell subtype, either no-target control or nonengaged^Enriched^ TEGs were designated as the root of the trajectory. To acquire comparable results from both Seurat and Monocle3 packages, the FastMNN batch-corrected UMAP coordinates were imported and used throughout the trajectory analysis in Monocle3. In IL17RB^−^CD4^+eff^ and IL17RB^+^CD4^+mem^ subtypes, Monocle identified no-target control cells as a separate partition. To have all cells along with a single pseudotime spectrum, we added maximum pseudotime values of no-target control T cells to pseudotime values of remaining cells in that subtype. For all TEG subtypes, significant dynamic genes along with the pseudotime trajectory were calculated and identified using Monocle3’s graph_test function, with 1 × 10^–20^ *q-*value as the significance cutoff. Afterwards, using both *k*-means clustering and visual inspection of gene behavior over the pseudotime, TEGs were clustered into subclusters of similar pattern (CL1–8; Fig. 5g). The expression profile of the genes, along with the pseudotime trajectory, was plotted using the package pheatmap^58^ using row-scaled (*z*-score) expression values. Smoothed gene behavior was calculated and visualized recruiting the gam smoothing function in the ggplot2 package^59^.

### Behavior signature inference over pseudotime

To align pseudotime inference with the different behavioral signatures that we identified with BEHAV3D, we built a probability map distribution for different behavioral signatures over the pseudotime based on the fundamental principle of transitivity of probabilistic distribution (Fig. 5f). We defined three states of cells quantified by different methods:

- Behavioral_signatures (*B*_sig_): (Static, Lazy, Medium scanner, Scanner, Super scanner, Tickler, Engager, Super engager). Behavioral signatures of cells identified by imaging (Fig. 5b).
- Experimental_engagement_state (Exp_eng_): (No-target control, Nonengaged, Nonengaged^enriched^, Engaged, Super engaged). Cell distribution among different experimental conditions (Fig. 5a).
- UMAP_cluster (*U*_cl_): (1…*X*). Cell assignment to distinct clusters grouping cells of similar gene expression. Shared nearest-neighbor, graph-based clustering was repeated several times using the Seurat package FindNeighbors and FindClusters functions with resolution in the range 1–7.

From these three different cell states, the following information was quantified:

- *p*(*B*_sig_|Exp_eng_): for each Experimental_engagement_state we quantified the probability distribution of each Behavioral_signature (Fig. 5f). This was achieved by reproducing the Experimental_engagement_states in silico on our imaging data. These values were calculated separately for CD4^+^ and CD8^+^ TEGs.
- *p*(Exp_eng_|*U*_cl_): for each UMAP_cluster, we quantified the probability of each Experimental_engagement_state belonging to this cluster.

Given these probabilities, we then quantified for each T cell the probability distribution of each unique Behavioral_signature in each UMAP_cluster using the equation:

$$p\left( {B_{\mathrm{sig}}{\mathrm{|}}U_{\mathrm{cl}}} \right) = \mathop {\sum }\limits_{{\mathrm{Exp}}_{\mathrm{eng}}} p\left( {B_{\mathrm{sig}}{\mathrm{|}}{\mathrm{Exp}}_{\mathrm{eng}}} \right) \times p\left( {{\mathrm{Exp}}_{\mathrm{eng}}{\mathrm{|}}U_{\mathrm{cl}}} \right)$$

As a result, each cell was assigned a certain probability distribution for different behavioral signatures. To refine the probability map, the same process was repeated for seven runs with different cluster sizes and final probability distributions were averaged per cell. Note that, for cells belonging to the No-target control Experimental_engagement_state, a Behavioral_signature called No-target control was assumed. Given that the nonengaged behavioral signatures (Static, Lazy, Slow scanner, Medium scanner, Super scanner) exhibited an identical probability map, their values were plotted together. For visualization purpose, extreme outlier values of skewed distributions were transformed to a maximal cutoff value. Based on the probability distribution of different behavioral signatures, pseudotime was divided into four stages—Baseline (no organoids), Environmental stimuli, Short engagement and Prolonged engagement—for each TEG subtype (CD8^+eff^, CD4^+eff^ and CD4^+mem^).

### Serial killer gene signature analysis

Genes of CL7 (Fig. 5g and Supplementary Tables 4 and 5) were analyzed to identify a unique signature for killer TEGs. Sixty-one of 83 genes comprising this cluster were common to TEGs incubated with 13T and 10T organoids and underwent extensive literature curation to identify those with a known role in T cell cytotoxicity, T cell biology (not related to cytotoxicity), morphological plasticity or other processes such as GTPase signaling, ribogenesis and transcriptional regulation.

### Cytotoxic in vivo T cell signature definition and projection on TEGs

To generate a signature gene set for cytotoxic CD8^+^ T cells in samples from patients with BC, we downloaded two publicly available datasets from GEO (accession nos. GSE114724 (ref.^40^) and GSE110686 (ref. ^41^)). Raw data were downloaded and analyzed with Seurat, using the same procedure utilized for TEG data processing. Clusters were identified and named using the marker genes defined in the study of Savas et al.^41^. From the study of Azizi et al.^40^, only TILs were used for analysis. Clusters were generated with a resolution of 0.9. For the Azizi and Savas studies, two marker gene lists were identified for cytotoxic CD8^+^ T cells (based on the 2,000 variable features and an average log(fold change) cut off of 0.3; Supplementary Table 6). The overall enrichment of the identified gene sets for each study was calculated using VISION^60^ and visualized on top of UMAP cell embeddings for each study. In addition, the overall enrichment of in vivo identified gene sets was projected on the UMAP of TEGs.

For the following methods we refer to Supplementary Protocols: primary DMG patient-derived lines and head and neck cancer PDO cultures, cell lines, WT1 T cells, ROR1 CAR T cells, flow cytometry analysis of NCAM1 and ROR1 expression, sorting of NCAM1^–/+^ TEGs, T cell serial killing capacity analysis, PDO bulk RNA-seq, SORT–seq sample preparation, SORT–seq library preparation and sequencing, mapping and quantification of SORT–seq data, SORT–seq and 10X Genomics data integration and TEG subpopulation analysis, differential gene expression analysis of TEGs cocultured with distinct PDO cultures and gene set enrichment analysis.

### Statistics and reproducibility

Statistical analysis was performed using either R or Prism v.7 software (GraphPad), and results are represented as mean ± s.e.m. unless indicated otherwise; *n* represents independent biological replicates. Two-tailed unpaired *t*-tests were performed between two groups unless indicated otherwise. Pearson correlation was used for paired comparison among three different readouts (IFN-γ production, cell viability and live imaging). For live-cell imaging, the increase in dead cell dye between the first and last time points was used as a measure. To compare tumor volumes in mice treated with TEGs or TEG001 mock cells, two-way analysis of variance (ANOVA) with repeated measures was performed. To compare frequencies of different behavioral signatures among PDOs, a Pearson’s chi-squared test was applied. To compare the percentage of dead organoids when TEGs were cocultured with different PDOs, one-way ANOVA followed by Bonferroni correction was performed. To estimate the change in correlation between PDO death dynamics and cumulative contact with TEGs for different behavioral signatures, data were fitted to a linear mixed model with experimental replicate as the random effect to account for variation between them. For cell type enrichment analysis of TEG first and second action after engagement, a hypergeometric test was used (Fisher’s exact test). For comparisons of percentages of distinct TEG subtypes in the same well (CD4^+^ versus CD8^+^ or NCAM^+^ versus NCAM), for each behavioral signature data were fitted to a linear regression model with each individual replicate set as the random effect to account for variation between them. For comparisons of percentages between different T cell lines (different wells), the standard deviation of the difference between mean cluster percentages for pairs of T cell lines was calculated by taking the square root of the sum of the variances of both separate distributions (Fig. 3j). For each fitted model, ANOVA was computed with an *F*-test. For comparison of IFN-β treatment, paired *t*-tests were performed. To ensure global TEG behavior atlas (Fig. 2a,b) reproducibility, we pooled 22 different imaging datasets comprising TEGs and LM1 cells cocultured with 13T or 100T organoids. Supplementary Table 8 summarizes the value of *n* per condition for Figs. 2b, 3f–j and 6e–g and includes statistical test details from Fig. 2f.

### Reporting summary

Further information on research design is available in the Nature Research Reporting Summary linked to this article.

## Online content

Any methods, additional references, Nature Research reporting summaries, source data, extended data, supplementary information, acknowledgements, peer review information; details of author contributions and competing interests; and statements of data and code availability are available at 10.1038/s41587-022-01397-w.

## Supplementary information

Supplementary Information Supplementary discussion, protocols, table legends and references.

Reporting Summary

Supplementary Table 1 Characteristics of organoid cultures derived from 14 patients with breast cancer.

Supplementary Table 2 DEG analyses between the sixth-highest versus lowest TEG-sensitive tumoroid cultures from Fig. 1d.

Supplementary Table 3 DEG analyses between different TEG subpopulations identified in Extended Data Fig. 6a.

Supplementary Table 4 List of genes clustered based on distinct expression dynamics in TEGs during 13T PDO exposure and targeting corresponding to Fig. 5g.

Supplementary Table 5 Conserved genes of the (serial) killer TEG signature corresponding to Fig. 5k. Genes with known or previously undescribed T cell function are highlighted in blue or green, respectively.

Supplementary Table 6 DEG analyses between different tumor-infiltrating lymphocyte populations (from Savas et al.^41^ or Azizi et al.^40^).

Supplementary Table 7 DEG analyses and common genes corresponding to Fig. 6 analyses.

Supplementary Table 8 Summary of replicates per condition or statistical test details for a selection of data panels.

Supplementary Video 1 Visual summary of the technology and main findings of the article.

## Data Availability

RNA-seq data of this study have been deposited in the Gene Expression Omnibus under accession no. GSE172325 (https://www-ncbi-nlm-nih-gov.ezproxy.u-pec.fr/geo/query/acc.cgi?acc=GSE172325). Imaging data used for the behavioral reference map have been deposited in the BioImage Achive under accession no. S-BIAD448 (https://www.ebi.ac.uk/biostudies/studies/s-biad448).

## Code availability

We provide the BEHAV3D framework as a compilation of R scripts on github (https://github.com/alievakrash/BEHAV3D).
