# Supplementary material for: Uncovering the mode of action of engineered T cells in patient cancer organoids
Source: Nat Biotechnol. 2022 Jul 25;41(1):60–9. doi: 10.1038/s41587-022-01397-w (PMC9849137; doi:10.1038/s41587-022-01397-w)
Supplement: Supplementary file 1 — Supplementary discussion, protocols, table legends and references. [file 41587_2022_1397_MOESM1_ESM.pdf]

nature portfolio

https://doi.org/10.1038/s41587-022-01397-w

Supplementary information

Uncovering the mode of action of engineered T cells in patient cancer organoids

In the format provided by the authors and unedited

Uncovering the mode of action of engineered T cells in patient cancer organoids

Dekkers, Alieva *et al.*

Supplementary Information

Supplementary Table legends

Supplementary Discussion

Supplementary Protocols

Supplementary References

Supplementary Table legends

Supplementary Table 1. Characteristics of organoid cultures derived from 14 breast cancer patients.

Supplementary Table 2. DEG analyses between the sixth highest versus lowest TEG-sensitive tumoroid cultures from Fig. 1d.

Supplementary Table 3. DEG analyses between different TEG subpopulation identified in Extended Fig. 6a.

Supplementary Table 4. List of genes clustered based on distinct expression dynamics in TEGs during 13T PDO exposure and targeting corresponding to Fig. 5g.

Supplementary Table 5. Conserved genes of the (serial) killer TEG signature corresponding to Fig. 5k. Genes with known or previously undescribed T cell function are highlighted in blue or green, respectively.

Supplementary Table 6. DEG analyses between different tumor-infiltrating lymphocytes populations from Savas et al.41 or Azizi et al.40.

Supplementary Table 7. DEG analyses and common genes corresponding to Fig. 6 analyses.

Supplementary Table 8. Summary of replicates per condition or statistical test details for a selection of data panels.

Supplementary Discussion

By applying BEHAV3D, we show that, through unbiased clustering of dynamic imaging features, differences in behavior between different engineered T cell products can be uncovered, as well as changes in behavior induced by the type of PDO. Using this pipeline, we report on the broad targeting potential of TEGs for BC, poorly permissive to current immunotherapies[61], thereby, providing evidence in favor of clinical potential against solid tumors, albeit with various responsiveness between individual donors. In addition, through behavior-guided transcriptomics we have generated, to our knowledge, the first molecular map underlying the behavioral landscape of immune cells targeted to solid tumors. By exploiting these results, we were able to design an optimal sequence of IFN-I and TEG combination therapy to boost TEG BC PDO targeting.

Different from recent studies that have mapped the activation trajectories of murine immune cells during viral infection[62], or human immune cells in normal physiology or cancer[63], we here reconstructed activation trajectories for engineered T cells and uniquely exploited dynamic imaging data revealing their single-cell behavior. This allowed us to dissect gene programs induced by environmental stimuli, versus induction by short or prolonged tumor engagement, and thereby identify the gene signature of TEGs that (serially) killed tumor cells. A preferred behavioral feature of T cell therapies[31-33] that we also observed in WT1 T cells and ROR1 CAR T cells. This (serial) killing signature includes genes not previously linked to T cell function, thereby providing opportunities to potentially engineer next generation T cells with potent serial killing capability. Furthermore, multiple genes in this signature are associated with morphological plasticity. Such plasticity may underlie the remarkable cellular extensions of serial-killing TEGs observed in our 3D imaging data. Using these protrusions, TEGs intercalated between tumor cells while sequentially killing multiple tumor cells in the PDO, suggesting that morphological plasticity may be an important attribute in the targeting of solid

tumors. In support of this, we observed similar morphological plasticity also in WT1 T cells and ROR1 CAR T cells targeting solid tumor PDOs, which rely on a different mode-of-recognition (tumor-specific antigen), compared to metabolome-sensing TEGs.

By linking T cell behavior to population phenotypes, we were able to show that profound tumor targeting, including serial killing, was a predominant feature of $CD8^+$ TEGs, making this subset the most attractive effector population for treatment. Yet, $CD4^+$ T cells, for which we detected a specific behavioral signature characterized by high movement and short organoid engagement, are thought to display an indispensable role in supporting the cytotoxic function and persistence of $CD8^+$ engineered T cells[64, 65]. Thus, the preferred product for clinical treatment, will depend on an optimal combination between $CD4^+$ and $CD8^+$ subsets. Moreover, we show that tumor-targeting by $CD8^+$ TEGs can be further enhanced by sorting $NCAM1^+$ cells, thereby providing proof-of-concept that the efficacy of an engineered T cell population can be improved through cell selection. Similar results could potentially be achieved through functional skewing or combination therapy, as we observed higher proportions of $NCAM1^+$ cells in TEGs expanded in the presence of IL-15, and recombinant IL-15 is already used in a human clinical trial as a combination partner to natural killer cell immunotherapy[66].

Type 1 IFNs have been described to be beneficial for the control of tumor growth, including in breast cancer, either by exerting direct antitumor effects[67], or by improving the response to therapies, such as chemotherapy and checkpoint inhibition[68, 69]. Yet, opposite roles in inducing treatment resistance have been described as well[42, 70, 71]. By using defined BC immune-organoid co-cultures, we have shown that an IFN-I signature intrinsic to tumor cells associates with TEG sensitivity, and that IFN-β primes tumor cells for more efficient targeting, rather than directly affecting TEG killing behavior. Thus, our data support the clinical use of IFN-I in combination with TEGs and possibly other cellular immunotherapies.

Adding to patient-specific drug responses observed in PDOs biobanks[14-18, 72], we show that not only killing efficacy, but also the underlying behavioral and molecular mechanisms of cellular immunotherapy differ between different PDO cultures. We even detected differences in killing dynamics between individual organoids belonging to the same PDO culture that appeared to arise from intrinsic biological differences between individual organoids. This demonstrates that our platform captures inter- and intra-patient heterogeneity, a major obstacle for treating solid tumors[73]. It is intriguing that gene signatures induced in TEGs upon organoid engagement were partly dictated by the type of PDO. In addition, the extent of IFN-β pre-treatment outcome on tumor targeting differed between PDOs, with the highly resistant BC culture 100T remaining unresponsive, whereas 34T displayed the highest (4-fold) increase in targeting. Together, these findings warrant caution regarding generalizing the outcome of immuno-oncology studies that use a single tumor model, and further supports the value of human organoid technology for development of personalized therapies.

Altogether, BEHAV3D combines organoid, imaging and sequencing technologies to offer a comprehensive platform that integrates multiple single-cell readouts, including tumor death dynamics, single-cell behavior and underlying transcriptomic profiling (**Supplementary Video 1**). BEHAV3D may thus contribute to efforts aimed at enhancing the efficacy of solid tumor-targeting by cellular therapies.

Supplementary Protocols

Primary DMG patient-derived lines and head and neck cancer PDO cultures

Primary DMG patient-derived lines were cultured as previously described[74-76]. In short, DMG organoids were resuspended in tumor stem media (TSM), consisting of 50% DMEM/F12 (Thermo Fisher Scientific) and 50% Neurobasal-A (Thermo Fisher Scientific) medium, supplemented with 0.1mg/ml primocin (Thermo Fisher), 10 mM HEPES, 1% GlutaMax, 1% sodium pyruvate, 1x MEM non-essential amino acids, 1x B27 supplement minus Vitamin A (all from Thermo Fisher), 20 ng/ml recombinant human EGF, 20 ng/ml recombinant human FGF-2, 10 ng/ml platelet-derived growth factor (PDGF)-AA, 10 ng/ml PDGF-BB (all from PeproTech) and 2 µg/ml heparin (StemCell Technologies), and seeded in 6-well plates for suspension culture (Greiner Bio-One). Medium was refreshed twice a week and cultures were passaged every 1-2 weeks using StemPro Accutase Cell Dissociation Reagent (Thermo Fisher) for dissociation. Organoids from a 7-day old culture were used for T cell co-culture.

Head and neck cancer PDOs were seeded in BME in uncoated 12-well plates (Greiner Bio-one) and cultured as described previously[77]. In short, adDMEM/F12+++ was supplemented with 1x B27 supplement (GIBCO), 1.25 mM n-Acetylcystein (Sigma), 10 mM nicotinamide (Sigma), 50 ng/ml human EGF, 10 ng/ml human FGF-10, 5 ng/ml human FGF-2 (all from PeproTech), 500 nM A83-01 (Tocris Biosciences), 1 µM PGE2 (Tocris Biosciences), 0.3 µM CHIR-99021 (Stemgent), 1 µM forskolin (R&D Systems), 4% RSPO3-Fc fusion protein conditioned medium and 4% Noggin-Fc fusion protein conditioned medium (made in house) for cultures H&N1,2 & 4[77]. For H&N3, adDMEM/F12+++ was supplemented with 1% Noggin-conditioned medium (U-Protein Express), 1x B27 supplement (GIBCO), 2.5 mM nicotinamide (Sigma), 1.25 mM n-Acetylcystein (Sigma, Cat# A9165), 10 µM ROCK inhibitor (Abmole), 500 nM A83-01 (Tocris), 10 µM forskolin (R&D Systems), 25 ng/ml FGF7

(Peprotech) and 1 μM p38 inhibitor SB202190 (Sigma) (medium “M5” from Lohmussaar et al.[78]). Organoids were passaged and grown for 5-7 days before use in co-culture.

Cell lines

Daudi (CCL-213)[6], HL60 (CCL-240)[8] and Phoenix-Ampho (CRL-3213) cell lines were obtained from ATCC. Phil Greenberg (Fred Hutchinson Cancer Research Center, Seattle, USA) kindly provided LCL-TM. Daudi, HL60 and LCL-TM cells were cultured in RPMI media supplemented with 10% fetal calf serum (FCS) and 1% pen/strep (all from Thermo Fisher). Phoenix-Ampho cells were cultured in DMEM medium (Thermo Fisher) supplemented with 10% FCS and 1% pen/strep. All cells were passaged for a maximum of 2 months, after which new seed stocks were thawed for experimental use. Stocks were re-authenticated by short tandem repeat profiling/karyotyping analysis provided by Eurofins Genomics in 2019 (Daudi), 2021 (LCL-TM and Phoenix-Ampho) and 2022 (HL-60), respectively. Furthermore, all cell lines were routinely verified by growth rate, morphology, and/or flow cytometry and tested negative for mycoplasma using MycoAlert Mycoplasma Kit. Peripheral blood mononuclear cells (PBMCs) were obtained from Sanquin Blood bank (Amsterdam, The Netherlands) and isolated using Ficoll gradient centrifugation methods from buffy coats.

WT1 T cells

HLA-A*0201-restricted WT1126-134-specific αβTCR[30] was transduced into αβ T cells as described[79]. In brief, Phoenix-Ampho packaging cells were transfected with gag-pol (pHIT60), env (pCOLT-GALV) and pBullet retroviral constructs, containing TCRβ-chain-IRES-neomycine or TCRα-chain-IRES-puromycin, using Fugene6 (Promega). PBMCs were preactivated, transduced and expanded as described above for TEGs, and selected with 800 μg/ml geneticin (Gibco) and 5 μg/ml puromycin (Sigma-Aldrich) for one week. $CD8^+$ TCR-

transduced T cells were isolated by MACS-sorting using CD8-microbeads (Miltenyi Biotec). Transduced T cells were then stimulated using a REP[8] as described above for TEGs. Transgenic TCR expression and purity of $CD8^+$ populations was routinely assessed by flow cytometry.

ROR1 CAR T cells

The ROR1-specific chimeric antigen receptor (CAR) T cell was previously described[80]. The CAR sequence was derived from patent WO2016187216 and cloned into the pCCL-cPPT-hPGK-GFP-bPRE4-SIN lentiviral transfer vector in place of the GFP sequence, which was derived from the pRRL-cPPT-hPGK-GFP-bPRE4-SIN plasmid[81]. Lentiviral particles were produced using standard calcium phosphate transfection (Clontech) of HEK-293T cells (ATCC CRL-3216) as described previously[82]. $CD8^+$ T cells were separated from cord blood by ficoll isopaque density gradient centrifugation (GE Healthcare Bio-Sciences AB) and magnetic bead separation (Miltenyi Biotech). Cells were expanded and transduced according to a previously established protocol[82]. Transduced cells were subsequently FACS sorted for CAR expression (anti-IgG1-FITC) on a BD FACSAria II, after which the pure CAR expressing T cell population was stimulated using a REP[8] as described above for TEGs.

Flow cytometry analysis of NCAM1 and ROR1 expression

PDOs were dissociated into single cells using TrypLE Express (Thermo Fisher) at 37°C. Single cells were washed and stained in flow cytometry staining buffer (2% fetal bovine serum (FBS), 1x PBS) with ROR1-APC (1:50; Biolegend) and LIVE/DEAD Fixable Near-IR Dead Cell Stain (1:1000; Thermo Fisher) for 30 minutes at 4°C, followed by fixation in 4% paraformaldehyde (PFA) for 10 minutes at 4°C. Data acquisition was performed on a CytoFlex LX system (Beckman Coulter). To compare NCAM1 expression on TEGs after expansion in the presence or absence of 5 ng/ml IL-15 (Miltenyi), cells were stained with anti-CD4-PerCp

(1:25; BD Biosciences), anti-CD8-V500 (1:50; BD Biosciences), anti-αβTCR-FITC (1:10; AntibodyChain), anti-pan γδTCR-PE (1:10; Beckman Coulter), and anti-NCAM1/CD56-FITC (1:20; BD Biosciences). Data was analyzed using FlowJo version 10.8 (BD Life Sciences).

Sorting of NCAM1$^{-/+}$ TEGs

CD8^{+} TEGs were harvested at day 8-10 of their REP cycle, stained in flow cytometry (FC) buffer (2% fetal bovine serum, 1x PBS) with Hilyte-488-conjugated NCAM1 nanobodies (1:400; QVQ) and LIVE/DEAD Fixable Near-IR Dead Cell Stain (1:1000; ThermoFisher) for 30 minutes at 4˚C and consecutively sorted using a SONY SH800S or a FACS Aria Cell Sorter (BD Biosciences) into NCAM1^{-} and NCAM^{+} populations. Cells were rested for 16 h in 'T cell culture medium' and then used for co-culture.

T cell serial killing capacity analysis

For accurate long-term (up to 24 h) T cell tracking, TEGs were plated at an E:T ratio of 1:25. Tracks were manually corrected where required. Tracks were divided into shorter subtracks of 160 minutes. Using the random forest classifier described above (**Extended Data Fig. 3d,e**), each subtrack was assigned to a behavioral signature. The following statistics were calculated for each type of behavioral signature (9 clusters): for continuous variables (square displacement; speed, T cell death) the mean, median and standard deviation of the upper quantile were calculated, and for discrete variables (organoid contact and interaction with T cells) the mean, cumulative mean, maximum and cumulative maximum were calculated. Principal component (PC) analysis was used to reduce the dimensionality. The top 5 PCs were used to classify the change in behavioral signature over time (**Extended Data Fig. 4b,c**). Equivalent to the approach that was used for the full tracks in **Fig. 2b**, we computed a cross-distance matrix based on the multivariate time-series data using the dynamic time warping

algorithm and performed k-means clustering in UMAP space. The change in behavioral signatures was represented in a time-series color plot where each row represents one cell track and the color codes for behavioral signature (**Extended Data Fig. 4c)**. The relative proportion of $CD4^+$ and $CD8^+$ TEGs in each cluster was calculated and plotted next to each long-term classification (**Extended Data Fig. 4c**).

To quantify the number of cells killed by a TEG we divided the killed volume by the average volume of a single 13T cell (2182 μm^3).

PDO bulk RNA sequencing

For bulk RNA sequencing characterization, RNA of PDOs grown in 'Type 1' culture medium was isolated according to the manufacturer's protocol using the RNeasy Mini Kit (QIAGEN). Quality and quantity of the RNA samples and the libraries were measured with Agilent's Bioanalyzer2100 and Invitrogen™ Qubit™ 3.0 Fluorometer. Quality control was done using FastQC, alignment has been done using STAR (https://github.com/alexdobin/STAR/releases/tag/STAR_2.4.2a) and reads have been mapped to the GRCh37 version of the human reference genome. Quality control on the bams was done using Picard. Read counts were generated with Htseq-count after which normalization was done using DESeq. RPKMs have been calculated with edgeR. For the library preparation the TruSeq Stranded mRNA Library Prep kit from Illumina was used. Sequencing was performed on the nextseq500 sequencer (also Illumina) with single-end 75bp reads. PDO cultures were ranked by responsiveness to TEGs (**Fig. 1d**) and differentially expressed genes between the 6 most TEG-sensitive and 6 least TEG-sensitive cultures were analyzed. Genes exhibiting a more than 4-fold expression change with an adjusted p-value <0.05 after multiple hypothesis testing correction were used as input gene set enrichment analysis.

SORTseq sample preparation

For sequencing of different behavior-enriched TEG populations (**Fig. 5a**), TEGs (>0,8x10^{6} per condition) were either (1) co-cultured with 13T PDOs (E:T of 1:3) and separated into organoid-engaged (*engaged*) and organoid non-engaged (*non-engaged*) populations by 2 slow-spin (30 rcf) centrifugation steps at 6 h co-culture, (2) co-cultured with 10T or 13T PDOs (E:T of 1: 3) and separated at 4 hrs into organoid-engaged and organoid non-engaged populations by a slow-spin (30 rcf) centrifugation step, co-cultured for another 2h with or without addition of fresh PDOs, again followed 2 slow-spin (30 rcf) centrifugation steps to obtain *non-engaged*^{Enriched} and *super-engaged* TEG populations, or (3) cultured for six hrs without addition of PDOs (*no target control*), using 12-wells culture plates (Thermo Fischer) and 'co-culture medium'. To create single-cell suspensions, conditions containing organoids (all '*engaged'* TEG conditions) were treated with TrypLE for seven minutes at 37°C and washed with adDMEM/F12+++. Cells were then stained in FC buffer (2% FCS in PBS) with anti-CD3-APC conjugated antibodies (1:80; BioLegend) and LIVE/DEAD Fixable Near-IR Dead Cell Stain (1:1000; ThermoFisher) for 30 minutes at 4°C and sorted into 384-wells SORTseq plates using a FACS Aria Cell Sorter (BD Biosciences) and directly stored at -80°C until further processing.

SORTseq library preparation and sequencing

All sorted plates were processed according to the CEL-Seq2 protocol with the total transcriptome amplification via poly-A RNA-capture, library preparation, and sequencing into Illumina sequencing libraries as previously described[83]. Paired-end sequencing (read1: 30 bp; read2: 120 bp) was used to sequence the prepared libraries using an Illumina NextSeq sequencer.

Mapping and quantification of SORTseq data

SORTseq data were mapped and reads were counted, using STAR version 2.6.1a on the Hg38p10 human genome (annotated with GenCode v26). Plate-QC was performed using the Sharq pipeline[84]. Cells with mitochondrial mRNA reads higher than 15%, ribosomal RNA content higher than 30%, or ERCC reads higher than 25% were excluded from the downstream analysis. Cells with fewer than 650 and higher than 4500 genes captured, and genes captured in fewer than 2 cells per plate were also excluded.

SORTseq and 10x genomics data integration and TEG subpopulation analysis

For analysis of TEGs not exposed to organoids (**Fig. 4i; Extended Data Fig. 4g,h**), 3 experimental replicates were used consisting of two datasets processed using SORT-seq and one dataset processed using 10x Genomics Chromium Single Cell 3′ gene-expression kit. SORTseq data was processed as described above. For the 10x dataset, (fresh, not co-cultured) TEGs were viability-enriched via FACS by DAPI staining (1:1000; Thermo Fischer) and loaded according to the standard protocol of the Chromium Single Cell 3′ Kit (v3). All the following steps were performed according to the standard manufacturer's protocol. The library was sequenced on an Illumina Novaseq S1-flowcell and 19,000 reads/cell were collected. Single-cell RNAseq data were mapped, and counts of molecules per barcode were quantified using the cellranger(3.1.0) 10x software package to map sequencing data to the GRCh38(3.0.0) reference transcriptome supplied by 10x. Cells with mitochondrial mRNA reads higher than 15% and with fewer than 200 or more than 5000 distinct genes were excluded from the downstream analysis. Data were normalized by sequencing depth, scaled to 10,000 counts, log-transformed, and regressed against the UMI-counts and percentage of mitochondrial mRNA using the ScaleData function of the Seurat package. For integration of the 10x genomics (n = 1) and SORTseq (n = 2) datasets, we used previously published Seurat v3 data anchor-based

integration[85]. Briefly, all three datasets were normalized using SCTtransform[86] followed by selection of 5000 features for downstream integration. Shared nearest neighbor graph-based clustering was done using Seurat package's FindNeighbors and FindClusters functions with a resolution of 0.8. For cell type identification marker genes for each cluster were calculated using the FindAllMarkers function and examined to profile marker genes that correspond to known cell types. Additional support for identifying cell subpopulations similitudes was achieved by analyzing the differentially expressed genes with a cell-type annotation tool[87].

Differential gene expression analysis of TEGs co-cultured with distinct PDO cultures

For comparison of TEGs targeting 10T or 13T PDOs (**Fig. 6a-c**), SORTseq dataset was used including TEGs from distinct Experimental engagement states: *Non-engaged^{Enriched}* and *super engager*. *No target control* TEGs were used as a control group. SORTseq data were mapped and quantified and visualized with UMAP as described above. Differential gene expression analysis was performed with the FindMarkers function from Seurat v3. Common and specific gene sets were filtered and visualized by Venn diagram with the VennDiagram package.

Gene set enrichment analysis

The functional enrichment analysis in this study for pathway and biological processes annotations for gene sets of interest was conducted using ToppFun on the ToppGene Suite[88] (**Fig. 1h, Extended Data Fig. 6c, Extended Data Fig. 8b**). An enrichment score was assigned based on gene enrichment ratio and log p value. For redundant annotations, the annotation with the highest gene enrichment ratio was selected.

Supplementary References

61. Esteva, F.J., Hubbard-Lucey, V.M., Tang, J. & Pusztai, L. Immunotherapy and targeted therapy combinations in metastatic breast cancer. *Lancet Oncol* **20**, e175-e186 (2019).
62. Abbas, A. et al. The activation trajectory of plasmacytoid dendritic cells in vivo during a viral infection. *Nat Immunol* **21**, 983-997 (2020).
63. Szabo, P.A. et al. Single-cell transcriptomics of human T cells reveals tissue and activation signatures in health and disease. *Nat Commun* **10**, 4706 (2019).
64. Guedan, S. et al. Enhancing CAR T cell persistence through ICOS and 4-1BB costimulation. *JCI Insight* **3** (2018).
65. Zhao, Z. et al. Structural Design of Engineered Costimulation Determines Tumor Rejection Kinetics and Persistence of CAR T Cells. *Cancer Cell* **28**, 415-428 (2015).
66. Cooley, S. et al. First-in-human trial of rhIL-15 and haploidentical natural killer cell therapy for advanced acute myeloid leukemia. *Blood Adv* **3**, 1970-1980 (2019).
67. Dunn, G.P. et al. A critical function for type I interferons in cancer immunoediting. *Nat Immunol* **6**, 722-729 (2005).
68. Borden, E.C. Interferons alpha and beta in cancer: therapeutic opportunities from new insights. *Nat Rev Drug Discov* **18**, 219-234 (2019).
69. Sistigu, A. et al. Cancer cell-autonomous contribution of type I interferon signaling to the efficacy of chemotherapy. *Nat Med* **20**, 1301-1309 (2014).
70. Benci, J.L. et al. Tumor Interferon Signaling Regulates a Multigenic Resistance Program to Immune Checkpoint Blockade. *Cell* **167**, 1540-1554 e1512 (2016).
71. Jacquelot, N. et al. Sustained Type I interferon signaling as a mechanism of resistance to PD-1 blockade. *Cell Res* **29**, 846-861 (2019).
72. Jacob, F. et al. A Patient-Derived Glioblastoma Organoid Model and Biobank Recapitulates Inter- and Intra-tumoral Heterogeneity. *Cell* **180**, 188-204 e122 (2020).
73. Yamamoto, T.N., Kishton, R.J. & Restifo, N.P. Developing neoantigen-targeted T cell-based treatments for solid tumors. *Nat Med* **25**, 1488-1499 (2019).
74. Monje, M. et al. Hedgehog-responsive candidate cell of origin for diffuse intrinsic pontine glioma. *Proc Natl Acad Sci U S A* **108**, 4453-4458 (2011).
75. Mount, C.W. et al. Potent antitumor efficacy of anti-GD2 CAR T cells in H3-K27M(+) diffuse midline gliomas. *Nat Med* **24**, 572-579 (2018).
76. Pericoli, G. et al. Integration of Multiple Platforms for the Analysis of Multifluorescent Marking Technology Applied to Pediatric GBM and DIPG. *Int J Mol Sci* **21** (2020).
77. Driehuis, E., Kretzschmar, K. & Clevers, H. Establishment of patient-derived cancer organoids for drug-screening applications. *Nat Protoc* **15**, 3380-3409 (2020).
78. Lohmussaar, K. et al. Patient-derived organoids model cervical tissue dynamics and viral oncogenesis in cervical cancer. *Cell Stem Cell* **28**, 1380-1396 e1386 (2021).
79. Stanislawski, T. et al. Circumventing tolerance to a human MDM2-derived tumor antigen by TCR gene transfer. *Nat Immunol* **2**, 962-970 (2001).
80. Hudecek, M. et al. The B-cell tumor-associated antigen ROR1 can be targeted with T cells modified to express a ROR1-specific chimeric antigen receptor. *Blood* **116**, 4532-4541 (2010).
81. Huston, M.W. et al. Correction of murine SCID-X1 by lentiviral gene therapy using a codon-optimized IL2RG gene and minimal pretransplant conditioning. *Mol Ther* **19**, 1867-1877 (2011).
82. Lo Presti, V. et al. Efficient lentiviral transduction method to gene modify cord blood CD8(+) T cells for cancer therapy applications. *Mol Ther Methods Clin Dev* **21**, 357-368 (2021).
83. Muraro, M.J. et al. A Single-Cell Transcriptome Atlas of the Human Pancreas. *Cell Syst* **3**, 385-394 e383 (2016).

84. Candelli, T. et al. Sharq, A versatile preprocessing and QC pipeline for Single Cell RNA-seq. *bioRxiv* (2018).
85. Stuart, T. et al. Comprehensive Integration of Single-Cell Data. *Cell* **177**, 1888-1902 e1821 (2019).
86. Hafemeister, C. & Satija, R. Normalization and variance stabilization of single-cell RNA-seq data using regularized negative binomial regression. *Genome Biol* **20**, 296 (2019).
87. Cao, Y., Wang, X. & Peng, G. SCSA: A Cell Type Annotation Tool for Single-Cell RNA-seq Data. *Front Genet* **11**, 490 (2020).
88. Kaimal, V., Bardes, E.E., Tabar, S.C., Jegga, A.G. & Aronow, B.J. ToppCluster: a multiple gene list feature analyzer for comparative enrichment clustering and network-based dissection of biological systems. *Nucleic Acids Res* **38**, W96-102 (2010).
