# Supplementary material for: Uncovering the mode of action of engineered T cells in patient cancer organoids
Source: Nat Biotechnol. 2022 Jul 25;41(1):60–9. doi: 10.1038/s41587-022-01397-w (PMC9849137; doi:10.1038/s41587-022-01397-w)
Supplement: Supplementary file 2 — Reporting Summary [file 41587_2022_1397_MOESM2_ESM.pdf]

Corresponding author(s): Anne Caroline Rios
Last updated by author(s): May 15, 2022

Reporting Summary

Nature Research wishes to improve the reproducibility of the work that we publish. This form provides structure for consistency and transparency in reporting. For further information on Nature Research policies, see our Editorial Policies and the Editorial Policy Checklist.

Statistics

For all statistical analyses, confirm that the following items are present in the figure legend, table legend, main text, or Methods section.

n/a	Confirmed	
☐	☒	The exact sample size (*n*) for each experimental group/condition, given as a discrete number and unit of measurement
☐	☒	A statement on whether measurements were taken from distinct samples or whether the same sample was measured repeatedly
☐	☒	The statistical test(s) used AND whether they are one- or two-sided<br>*Only common tests should be described solely by name; describe more complex techniques in the Methods section.*
☒	☐	A description of all covariates tested
☐	☒	A description of any assumptions or corrections, such as tests of normality and adjustment for multiple comparisons
☐	☒	A full description of the statistical parameters including central tendency (e.g. means) or other basic estimates (e.g. regression coefficient) AND variation (e.g. standard deviation) or associated estimates of uncertainty (e.g. confidence intervals)
☐	☒	For null hypothesis testing, the test statistic (e.g. *F*, *t*, *r*) with confidence intervals, effect sizes, degrees of freedom and *P* value noted<br>*Give P values as exact values whenever suitable.*
☒	☐	For Bayesian analysis, information on the choice of priors and Markov chain Monte Carlo settings
☒	☐	For hierarchical and complex designs, identification of the appropriate level for tests and full reporting of outcomes
☐	☒	Estimates of effect sizes (e.g. Cohen's *d*, Pearson's *r*), indicating how they were calculated

Our web collection on statistics for biologists contains articles on many of the points above.

Software and code

Policy information about availability of computer code

Data collection	Zeiss Zen Black Edition (v2.3)
Data analysis	For 3D visualization, cell segmentation and extraction of statistics, time-lapse movies were processed with commerical software Imaris (Oxford Instruments), versions 9.2 to 9.5. Exported statistics were processed and analyzed with R (versions R 3.5.2 to R4.0.5) using a custom build framework, called BEHAV3D. We provide the BEHAV3D framework as a compilation of R scripts and example datasets on github (https://github.com/alievakrash/BEHAV3D). The following R packages were used for image and sequencing analysis: data.table_1.14.2; Deseq2_1.30.1; dplyr_1.0.7; dtw_1.22-3; eulerr_6.1.1; gganimate_1.0.7; ggplot2_3.3.5; ggpubr_0.4.0; ggrepel_0.9.1; gridExtra_2.3; hypergeo_1.2-13; kmodR_0.1.0; lme4_1.1-29; lmerTest_3.1-3; MESS_0.5.7; monocle3_1.0.1; nlme_3.1-157; openxlsx_4.2.4; patchwork_1.1.1; patternplot_1.0.0; pheatmap_1.0.12; plotly_4.10.0; plyr_1.8.7; png_0.1-7; purrr_0.3.4; RColorBrewer_1.1-3; readxl_1.4.0; reshape2_1.4.4; rgeos_0.5-9; scales_1.2.0; Seurat_4.0.4; Seurat_Object_4.0.2 ; SeuratWrappers_0.3.0; sp_1.4-7 ; spatstat_2.3-4 ; tidyr_1.2.0; tidyverse_1.3.1; umap_0.2.8.0; VennDiagram_1.7.3; viridis_0.6.2; VISION_2.1.0; xlsx_0.6.5; zoo_1.8-10.

For manuscripts utilizing custom algorithms or software that are central to the research but not yet described in published literature, software must be made available to editors and reviewers. We strongly encourage code deposition in a community repository (e.g. GitHub). See the Nature Research guidelines for submitting code & software for further information.

Data

Policy information about availability of data

All manuscripts must include a data availability statement. This statement should provide the following information, where applicable:

- Accession codes, unique identifiers, or web links for publicly available datasets
- A list of figures that have associated raw data
- A description of any restrictions on data availability

Data availability
RNA sequencing data of this study have been deposited in the Gene Expression Omnibus (GEO), under accession number GSE172325 (https://www-ncbi-nlm-nih-gov.ezproxy.u-pec.fr/geo/query/acc.cgi?acc=GSE172325). Imaging data used for the behavioral reference map have been deposited in the BioImage Achive (https://www.ebi.ac.uk/bioimage-archive/), under accession number TMP_1652359615399. Other imaging datasets can be provided on request.

Field-specific reporting

Please select the one below that is the best fit for your research. If you are not sure, read the appropriate sections before making your selection.

☒ Life sciences ☐ Behavioural & social sciences ☐ Ecological, evolutionary & environmental sciences

For a reference copy of the document with all sections, see nature.com/documents/nr-reporting-summary-flat.pdf

Life sciences study design

All studies must disclose on these points even when the disclosure is negative.

Sample size	For each experiment we empirically determined the sample size sufficient for reproducibility. Sample sizes were at least n=3 independent biological replicates. For animal experiments we calculated sample size by power calculation (power of 80% and significance of 0.05) by using 1-way ANOVA and taking treatment effects of previous studies into account.
Data exclusions	No experimental replicates were excluded. For SORTseq experiments cells with mitochondrial mRNA reads higher than 15%, ribosomal RNA content higher than 30%, or ERCC reads higher than 25% were excluded from the downstream analysis. Cells with fewer than 650 and higher than 4500 genes captured, and genes captured in fewer than 2 cells per plate were also excluded. For 10x experiments cells with mitochondrial mRNA reads higher than 15% and with fewer than 200 or more than 5000 distinct genes were excluded from the downstream analysis.
Replication	Information about the replicate numbers are detailed in the figure legends and in Supplementary Table 8.
Randomization	For animal experiment in Figure 1, mice were randomized with equal distribution by age and initial weight measured on day 0 and divided into 10–15 mice per group. In all other experiments samples were randomly allocated to groups.
Blinding	For animal experiment the investigators where blinded to the group allocation during data collection. For all other experiments blinding was not considered relevant.

Reporting for specific materials, systems and methods

We require information from authors about some types of materials, experimental systems and methods used in many studies. Here, indicate whether each material, system or method listed is relevant to your study. If you are not sure if a list item applies to your research, read the appropriate section before selecting a response.

Materials & experimental systems

n/a	Involved in the study	
☐	☒	Antibodies
☐	☒	Eukaryotic cell lines
☒	☐	Palaeontology and archaeology
☐	☒	Animals and other organisms
☒	☐	Human research participants
☒	☐	Clinical data
☒	☐	Dual use research of concern

Methods

n/a	Involved in the study	
☒	☐	ChIP-seq
☐	☒	Flow cytometry
☒	☐	MRI-based neuroimaging

Antibodies

Antibodies used	Anti-CD3-APC conjugated antibodies (Clone SK7; BioLegend; 344811)<br>anti-pan gamma delta TCR-PE (clone IMMU510; Beckman Coulter; B49176)

	anti-alpha beta TCR-FITC (clone IP26; eBioscience;11-9986-42)<br>anti-CD8-PerCP-Cy5.5 (clone SK1; Biolegend; 344709)<br>anti-CD4-APC (clone RPA-T4; Biolegend; 300514)<br>Hilyte-488-conjugated NCAM1 nanobodies (llana VHH; QVQ)
Validation	Anti-CD3-APC (https://www.biolegend.com/en-us/products/apc-anti-human-cd3-antibody-6780?GroupID=BLG5900)<br>Cited in e.g. Wadley AJ, et al. 2018. MethodsX and Claireaux M, et al. 2018. MBio. 9:e00317<br>We validated negative staining on BC organoid cells (CD3 neg) and positive staining on TEG(CD3+)<br><br>Anti-pan gamma delta TCR-PE, anti-alpha beta TCR-FITC, anti-CD8-PerCP-Cy5.5 and anti-CD4-APC were validated by Flow cytometry by comparing transduced (gamma detla TCR pos) versus untransduced T cells (gamma detla TCR neg) and by MACS of CD8+ and CD4 + TEGs. Other study that sucessfully used these antibodies: Marcu-Malina, V. et al. Redirecting αβ T cells against cancer cells by transfer of a broadly tumor-reactive γδT-cell receptor. Blood 118, 50–59 (2011), Vyborova, A. et al. γ9δ2T cell diversity and the receptor interface with tumor cells. J. Clin. Invest. 130(9):4637–4651 (2020), Gründer, C. et al. γ9 and δ2CDR3 domains regulate functional avidity of T cells harboring γ9δ2TCRs. Blood 120, 5153–5162 (2012).<br><br>Hilyte-488-conjugated NCAM1 nanobodies were validated by imaging of NCAM1- and NCAM1+ organoid cultures

Eukaryotic cell lines

Policy information about cell lines

Cell line source(s)	Daudi; https://www.atcc.org/products/ccl-213<br>HL60; https://www.atcc.org/products/ccl-240<br>Phoenix-Ampho; https://www.atcc.org/products/crl-3213
Authentication	Authenticated by short tandem repeat profiling/karyotyping/isoenzyme analysis
Mycoplasma contamination	All cell lines were routinely verified by growth rate, morphology, and/or flow cytometry and tested negative for mycoplasma using MycoAlert Mycoplasma Kit
Commonly misidentified lines (See ICLAC register)	No commonly misidentified lines were used in this study

Animals and other organisms

Policy information about studies involving animals; ARRIVE guidelines recommended for reporting animal research

Laboratory animals	NOD.Cg-PrkdcscidIl2rgtm1Wjl/SzJ (NSG) mice were purchased from Charles River Laboratories (France). Experiments were conducted with permission from the Animal Welfare Body Utrecht (4288-1-08 and 4288-1-09). Mice were housed at 45-65% humidity and 12 hrs light and 12 hours dark per day, in sterile conditions using an individually ventilated cage (IVC) system and fed with sterile food and water.
Wild animals	No wild animals were used in this study
Field-collected samples	No field collected samples were used in the study.
Ethics oversight	Experiments were conducted in accordance with Institutional Guidelines under acquired permission from the local Ethical Committee and as per current Dutch laws on Animal Experimentation

Note that full information on the approval of the study protocol must also be provided in the manuscript.

Flow Cytometry

Plots

Confirm that:

☒ The axis labels state the marker and fluorochrome used (e.g. CD4-FITC).

☒ The axis scales are clearly visible. Include numbers along axes only for bottom left plot of group (a 'group' is an analysis of identical markers).

☒ All plots are contour plots with outliers or pseudocolor plots.

☒ A numerical value for number of cells or percentage (with statistics) is provided.

Methodology

Sample preparation	Human PBMCs from healthy donors were pre-activated with anti CD3 (30 ng/mL; Orthoclone OKT3; Janssen-Cilag) and IL-2 (50 IU/mL; Proleukin, Novartis) and subsequently transduced twice with viral supernatant (gdTCR) within 48 hrs in the

presence of 50 IU/mL IL-2 and 6 mg/mL polybrene (Sigma-Aldrich). TCR-transduced T cells were expanded by stimulation with anti-CD3/CD28 Dynabeads (500,000 beads/106 cells; Life Technologies) and IL-2 (50 IU/mL). Thereafter, TCR-transduced T cells were depleted of the non-engineered T cells (abTCR+) using MACS. In order to separate CD4+ and CD8+ TEGs and LM1s, we performed positive selection using either CD4 or CD8 Microbeads (Miltenyi Biotech) following manufacturer's instructions. TEGs were stimulated biweekly by using the REP protocol. In order to monitor the purity of CD4+ and CD8+ TEGs, cells were analyzed by flow cytometry weekly prior to functional assays by using anti-pan gdTCR-PE (Beckman Coulter), anti-abTCR-FITC (eBioscience), anti-CD8-PerCP-Cy5.5 (Biolegend) and anti-CD4-APC (Biolegend) antibodies.

Sorting of NCAM1-/+ TEGs
CD8+ TEGs were harvested at day 8-10 of their REP cycle, stained in flow cytometry (FC) buffer (2% fetal bovine serum, 1x PBS) with Hilyte-488-conjugated NCAM1 nanobodies (1:400; QVQ) and LIVE/DEAD Fixable Near-IR Dead Cell Stain (1:1000; ThermoFisher) for 30 minutes at 4C̃ and consecutively sorted using a SONY SH800S or a FACS Aria Cell Sorter (BD Biosciences) into NCAM1- and NCAM+ populations. Cells were rested for 16 h in 'TEG culture medium' and then used for co-culture.

SORTseq sample preparation
For sequencing of different behavior-enriched TEG populations (Fig. 4a), TEGs (>0,8x106 per condition) were either (1) co-cultured with 13T PDOs (E:T of 1:3) and separated into organoid-engaged (engaged) and organoid non-engaged (non-engaged) populations by 2 slow-spin (30 rcf) centrifugation steps at 6 h co-culture, (2) co-cultured with 10T or 13T PDOs (E:T of 1: 3) and separated at 4 hrs into organoid-engaged and organoid non-engaged populations by a slow-spin (30 rcf) centrifugation step, co-cultured for another 2h with or without addition of fresh PDOs, again followed 2 slow-spin (30 rcf) centrifugation steps to obtain non-engagedEnriched and super-engaged TEG populations, or (3) cultured for six hrs without addition of PDOs (no target control), using 12-wells culture plates (Thermo Fischer) and 'co-culture medium'. To create single-cell suspensions, conditions containing organoids (all 'engaged' TEG conditions) were treated with TrypLE for seven minutes at 37°C and washed with adDMEM/F12+++. Cells were then stained in FC buffer (2% FCS in PBS) with anti-CD3-APC conjugated antibodies (1:80; BioLegend) and LIVE/DEAD Fixable Near-IR Dead Cell Stain (1:1000; ThermoFisher) for 30 minutes at 4°C and sorted into 384-wells SORTseq plates using a FACS Aria Cell Sorter (BD Biosciences) and directly stored at -80°C until further processing.

Instrument

SONY SH800S or a FACS Aria Cell Sorter (BD Biosciences)

Software

FlowJo https://www.flowjo.com/

Cell population abundance

NCAM sorts: After sort TEGs were rested for 16 h in 'TEG culture medium' and labeled with with Hilyte-488-conjugated NCAM1 nanobodies (1:400; QVQ) and NCAM1 expression in positive and negative populations was confirmed using confocal microscopy (>100 % of cells positive as expected).

SORTseq: CD3+ cells were sorted and RNA sequenced; CD3 RNA expression was confirmed in the sequencing data set (>100% of cells)

Gating strategy

Cells (FCS/SSC) --> single cells (FSC-H/FSC-A) --> living cells (dead cell dye negative) --> NCAM+ OR CD3+ OR abTCR+ OR gdTCR + OR CD8+ OR CD4+

negative and positive gates were based on no antibody negative control samples

☒ Tick this box to confirm that a figure exemplifying the gating strategy is provided in the Supplementary Information.
